# Differential Item Functioning (DIF) in composite health measurement scale: Recommendations for characterizing DIF with meaningful consequences within the Rasch model framework

**DOI:** 10.1371/journal.pone.0215073

**Published:** 2019-04-09

**Authors:** Alexandra Rouquette, Jean-Benoit Hardouin, Alexis Vanhaesebrouck, Véronique Sébille, Joël Coste

**Affiliations:** 1 AP-HP, Bicêtre Hôpitaux Universitaires Paris Sud, Public Health and Epidemiology Department, Le Kremlin-Bicêtre, France; 2 CESP, INSERM, Université Paris-Sud, Université Versailles Saint-Quentin, Université Paris-Saclay, Paris, France; 3 EA 4360—APEMAC, Université de Lorraine, Université Paris-Descartes, Paris, France; 4 INSERM U1246 –SPHERE « MethodS in Patients-centered outcomes and HEalth REsearch », University of Nantes, University of Tours, Nantes, France; 5 Biostatistics and Methodology Unit, CHU de Nantes, Nantes, France; 6 AP-HP, Hôtel-Dieu Hospital, Biostatistics and Epidemiology Department, Paris, France; University of Copenhagen, DENMARK

## Abstract

**Objective:**

The aims were to review practices concerning Differential Item Functioning (DIF) detection in composite measurement scales, particularly those used in health research, and to provide guidance on how to proceed if statistically significant DIF is detected.

**Methods:**

This work specifically addressed the Rasch model which is the subject of growing interest in the field of health owing to its particularly advantageous properties. There were three steps: 1) Literature review to describe current practices; 2) Simulation study to determine under which conditions encountered in health research studies can erroneous conclusions be drawn from group comparisons when a scale is affected by DIF but which is not considered; 3) Based on steps 1 and 2, formulation of recommendations that were subsequently reviewed by leading internationally recognized experts.

**Results:**

Four key recommendations were formulated to help researchers to determine whether statistically significant DIF is meaningful in practice, according to the kind of DIF (uniform or non-uniform) and the DIF effect size.

**Conclusion:**

This work provides the first recommendations on how to deal in practice with the presence of DIF in composite measurement scales used in health research studies.

## 1. Introduction

Other than some purely descriptive studies, almost all health research studies include group comparisons: typical study designs involve a primary outcome measured in every subject, whose occurrence (if categorical) or mean (if continuous) is compared between groups defined by a characteristic or exposure of interest. More complex designs require multivariate analyses or subgroup analyses. To be accurate, the measurement of the outcome must be valid in all groups studied. Otherwise, the difference (or the absence of difference) observed between groups may be, partly or totally, an artifact due to the measurement instrument not being valid in one or several groups. Accurate group comparisons require measurement invariance: “the measuring device should function in the same way across varied conditions, so long as those varied conditions are irrelevant to the attribute being measured” [1].

Composite measurement scales are used in epidemiological studies to measure complex health related constructs, including quality of life, depression and satisfaction with care. They consist of items that assess the various dimensions of the construct to be measured. Several standards and guidelines have been published concerning their validation and measurement invariance is considered to be essential [2–6]. Statistical methods for assessing measurement invariance rely, directly or indirectly, on the “matching principle” [7]. Measurement invariance holds if two subjects being identical (thus “matched”) on the measured construct but from two different groups (males and females, for example) have the same probability of giving any particular answer to any item of the scale [1,8]. Classical statistical methods, termed “observed variable methods”, use the total score (sum of the responses to the items of the dimension studied) as a proxy for the position of the subjects on the measured construct and thus, as the matching variable. Then, logistic regression, for example, is used to model the probability of the response to a binary item as a function of the group membership and of the matching variable [9]. If the regression coefficient related to the group membership is statistically significant, the probability of responding positively to this item is significantly different between the groups whatever the level on the measured construct. This phenomenon is termed differential item functioning (DIF): the item “functions” differently in the groups to be compared [8,10]. Two kinds of DIF can be distinguished: uniform if the relation between the group and the response to the item is identical at every level of the matching variable; otherwise DIF is non-uniform [8]. If DIF, uniform or non-uniform, is present in a scale, measurement invariance does not hold and group comparisons may be inaccurate.

Other observed variable methods to detect DIF have been developed (Mantel-Haenszel method, standardization method, etc.) but have drawbacks, particularly the use of a proxy as the matching variable, motivating the development of more modern DIF detection methods [1,11–13]. Measurement invariance can be examined through item response theory (IRT) or confirmatory factor analysis (CFA) in which latent variables are used to represent the construct to be measured: there is DIF if one or several parameters of the function relating the latent variable to the observations (responses to the scale items) are different across the groups compared [14]. Latent or observed variable methods to detect DIF all involve statistical tests, whose results are strongly influenced by sample size. With sufficiently large samples, DIF would be detected in all items of all scales [14]. Thus, rather than “Is there DIF in the scale?” the pertinent question is: “In what conditions does statistically significant DIF have significant consequences on the conclusions drawn from group comparison?”.

The Rasch model is often used to analyze questionnaire data in health research studies [15]. It expresses the probability that an individual will respond positively to a binary item as a function of his/her level on the latent variable (termed latent trait) and of an item parameter [16,17]. The Rasch model (and its extensions for polytomous items) is more convenient than more complex IRT models, to detect DIF because it allows studying the variations of the item parameter across groups without influence from additional parameters (discrimination parameter, guessing parameter, etc.). Also, it provides a straightforward DIF effect-size: the group difference in the item parameter [1]. This DIF effect-size can be used to characterize the magnitude of significant DIF within a scale: for example, a group difference in the item parameter higher than 0.5 logit is often used as a criterion for a meaningful DIF [12,18].

The purpose of this paper was to review practices concerning DIF detection and handling in composite measurement scales used in health research studies and to provide guidance on how to proceed when DIF is statistically significant. We focused on the Rasch model which is the subject of growing interest in the field of health owing to its particularly advantageous properties. As theoretical background on meaningfulness of consequences of DIF is scarce in literature, this work was designed to gather information from various sources. It included three steps:

1. Literature review: description of current practices in health research studies concerning detection and handling of DIF in a scale, with a particular attention to the use of Rasch models in this context to bring out typical scenarios studied within the Rasch framework;
2. Simulation study: results from the literature review were used to enrich a recently published simulation study aiming to determine the conditions in which erroneous conclusions may be drawn from group comparisons in health research studies, when DIF is present within a scale [19];
3. Recommendations formulation: the literature review and simulation study were exploited to establish recommendations that were then reviewed by a panel of experts.

## 2. Literature review

### 2.1. Objectives

To describe current practice in health research studies to detect and handle DIF in a scale, with particular focus on the Rasch framework.

### 2.2. Methods

Articles written in English and published between November 11, 2013 and November 10, 2014 were selected from Medline using the following query to search title, abstract and keyword fields: (*“differential item functioning”* or *“item bias”* or *“measurement invariance”* or *“factorial invariance”* or *“measurement equivalence”)*. The Medline database was chosen as the most appropriate to describe current practices in health research studies and the time frame restricted to one year as the number of published articles allowed the whole of known DIF detection methods to be covered. As we focused on current practices, only articles describing empirical studies in which measurement invariance of composite measurement scale(s) was evaluated were included (reviews and editorials not included, as well as studies concerning item banks and not scales). Articles were excluded if DIF was only evaluated across collection times (longitudinal DIF).

The DIF detection method used and the research field (education, psychology and psychiatry, quality of life, medical disciplines) to which the scale was related were recorded for each article. Characteristics of the scales studied (number of dimensions, items and response categories per item) and practices for DIF assessment in articles using IRT for DIF detection were identified: the stated study aim, characteristic(s) determining the groups considered for DIF and sample size. Practices for DIF assessment included: assessment of non-uniform DIF, use of criteria based on DIF effect-size to classify statistically significant DIF as meaningful or not, empirical evaluation of the impact of the presence of DIF within the scale, practices and/or recommendations produced by authors on how to handle DIF detected.

Two of the investigators (AV and AR) did the data extraction and literature analysis: AV extracted all the data and AR checked studies in which IRT-based methods were used or in which data extraction was considered challenging by AV. All disagreements were resolved by discussion between AV and AR. Descriptive analysis was performed for quantitative data and qualitative content analysis was performed for studies with statistically significant DIF to reveal the methods employed to evaluate the impact of DIF and the practices and/or recommendations for its management.

### 2.3. Results

#### 2.3.1. Frameworks and scale domains in studies with DIF assessment

**Fig 1:**
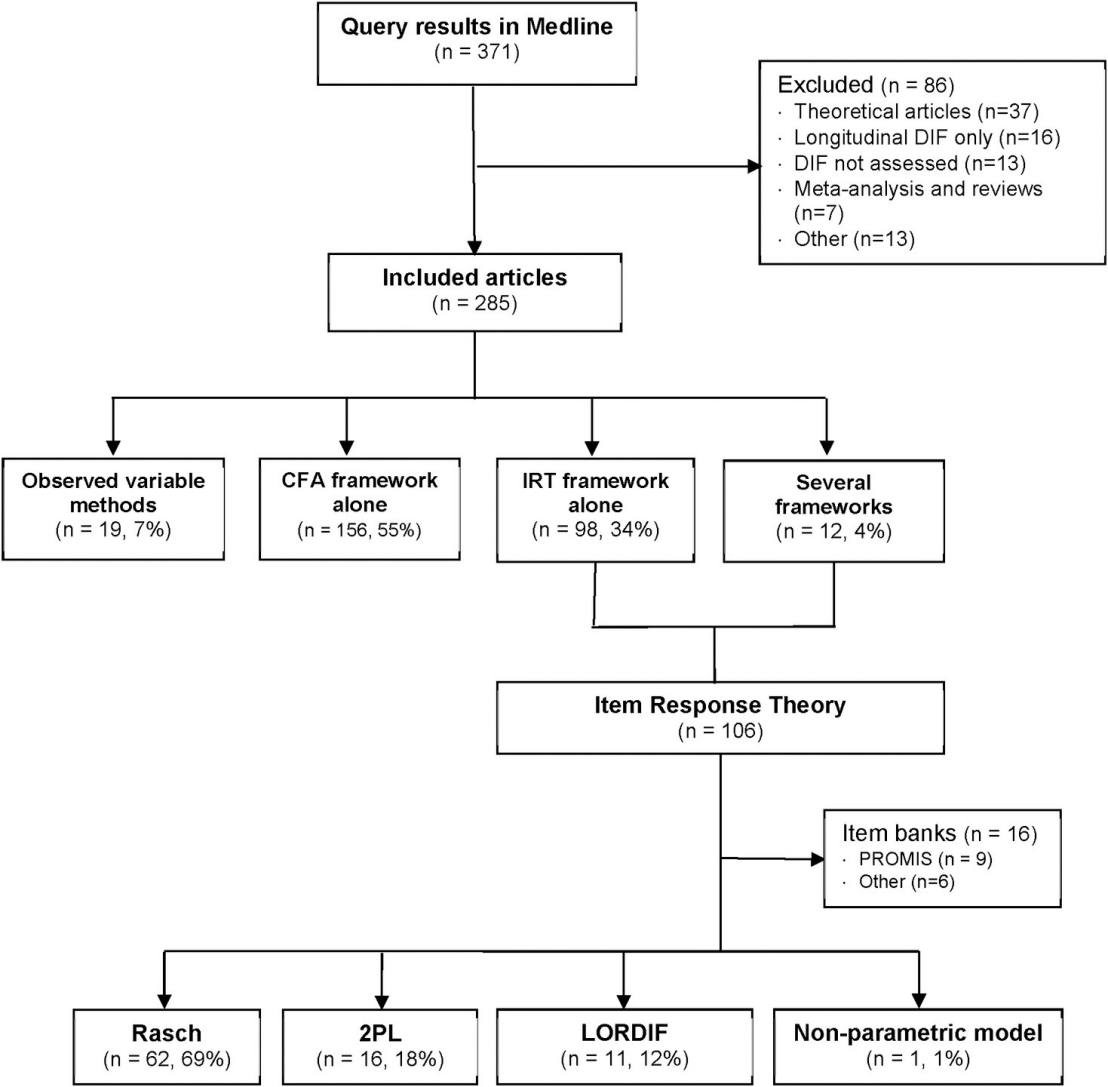
Flowchart of the selection of articles reporting detection of Differential Item Functioning (DIF). DIF: Differential Item Functioning, CFA: Confirmatory Factorial Analysis, IRT: Item Response Theory, PROMIS: Patient Reported Outcomes Measurement Information System, 2PL: Two Parameters Logistic model, LORDIF: Logistic Ordinal Regression Differential Item Functioning using IRT [20].

A total of 285 articles met the inclusion criteria (Fig 1 and S1 List). In 156 (55%) articles, measurement invariance was assessed within the CFA framework only, in 98 (34%) it was assessed within the IRT framework only, and in 19 (7%) articles only observed variable methods were used (12 logistic regression, 4 Mantel-Haenszel, 3 others). In the remaining 12 (4%) articles, several frameworks were used: both CFA and IRT frameworks in six, and observed variable methods plus CFA or IRT frameworks in six. The research field most frequently studied was “psychology and psychiatry” (n = 172, 60%) followed by “medical disciplines” (n = 60, 21%), “quality of life” (n = 35, 12%) and “education” (n = 18, 6%). The CFA framework was the most widely used in the “psychology and psychiatry” field (n = 126, 73%), the IRT framework was more frequent in “quality of life” (n = 20, 57%) and “medical disciplines” fields (n = 36, 60%). IRT (n = 7, 39%) and CFA (n = 8, 44%) frameworks were similarly used in the “education” field.

#### 2.3.2. Description of studies using the Rasch framework

Within articles using an IRT framework, 16 were excluded from further analysis as DIF was assessed in items from item banks and not in composite measurement scales (Fig 1). The aims of the other 90 studies were: development of a new scale (n = 17, 19%), evaluation of the psychometric properties of an existing scale (n = 61, 68%), cross-cultural adaptation (n = 7, 8%) and epidemiological study in which the scale was used to measure the outcome (n = 5, 6%). Rasch framework models were used the most (n = 62, 69%) followed by the two-parameter logistic model and its extensions for polytomous items (2PL, n = 16, 18%). In 11 (12%) articles, the DIF detection method combined logistic ordinal regression with IRT estimates of the latent trait as matching variable (LORDIF: Logistic Ordinal Regression Differential Item Functioning using IRT [20–22]). The median number of characteristics determining the groups considered for DIF in the same study was two (1^st^-3^rd^ quartiles: 1–4), and the maximum was nine. Sex (n = 59, 66%) and age (n = 53, 59%) were the most studied, followed by culture, country or ethnicity (n = 18, 20%) and education (n = 12, 13%).

Thirty-four (49%) of the 70 scales studied in the 62 articles using Rasch framework models were multidimensional, and within a dimension, the median number of items was nine (1^st^-3^rd^ quartiles: 6–15). The median number of dimensions in multidimensional scales was four (1^st^-3^rd^ quartiles: 2–5). Items were polytomous in 60 (86%) scales with a median number of response categories of five (1^st^-3^rd^ quartiles: 4–5). Table 1 summarizes practices concerning DIF assessment using the three main methods (Rasch, 2PL, LORDIF). The sample size was generally smaller for Rasch (median = 300, 1^st^-3^rd^ quartiles: 166–610) than the other two (median>950) methods. Non-uniform DIF was assessed in fewer than a quarter of the studies using the Rasch framework or the 2PL model whereas it was assessed in most articles using the LORDIF method (n = 7, 63%). Criteria based on DIF effect-size were used in fewer than one third of articles using the Rasch framework (n = 18, 30%) and various thresholds were used to consider DIF as meaningful: group difference in item parameter higher than 0.43 logit (n = 2) or 0.5 logit (n = 12) or 0.64 logit (n = 1) or 1 logit (n = 2). DIF effect-size criteria were used in most articles using LORDIF methods (n = 9, 82%, mostly based on change of the logistic regression parameter or pseudo R^2^ between two nested models) but in fewer of the articles using 2PL models (n = 3, 19%).

**Table 1:** Practices related to the Differential Item Functioning (DIF) detection and results within the item response theory framework.

	Rasch 62 articles	2PL 16 articles	LORDIF 11 articles
Sample size*	300 (166–610)	964 (762–2429)	1082 (573–2520)
Non-uniform DIF assessed, n (%)	13 (21%)	4 (25%)	7 (63%)
Use of DIF effect-size, n (%)	18 (30%)	3 (19%)	9 (82%)
Statistically significant DIF detected, n (%)	49 (79%)	14 (88%)	8 (73%)

* Median (1^st^ quartile– 3^rd^ quartile), 2PL: two parameters logistic, LORDIF: Logistic Ordinal Regression Differential Item Functioning using Item Response Theory [20]

Most studies concluded that DIF was present within the scale: 49 (79%) articles in the Rasch subgroup, 14 (87%) articles in the 2PL subgroup and eight (73%) articles in the LORDIF subgroup. In 18 (25%) of these 71 articles, the impact of the DIF was evaluated empirically and was considered to be meaningful in only four (22%) articles (S1 Table). Modifications to the scale or recommendations for its use in practice were suggested by the authors in 26 (37%) of the articles in which statistically significant DIF was found.

## 3. Simulation study

### 3.1. Objective

To determine the conditions in which erroneous conclusions may be drawn from group comparisons due to DIF within a scale in health research studies.

The methods and results described below are from a published study in which 632 realistic scenarios encountered in health research studies were simulated [19]. These scenarios are all based on a typical study which aims to detect a difference between two groups on a construct measured on a latent trait using a composite measurement scale. Factors (characteristics of the sample, of the scale and of items exhibiting DIF) which can be manipulated in the simulation model are described in Table 2. Their effect on the breadth of the bias resulting from the estimation of the difference between the two groups with no consideration of the presence of DIF in the scale was studied. For the study we describe here, 90 scenarios were added following the literature review which revealed that various commonly encountered scenarios had not been investigated in the previous study.

**Table 2:** Factors manipulated in the simulation study and values studied in the four kinds of Differential Item Functioning (DIF).

Factor	Values studied			
	Uniform DIF	Balanced non-uniform DIF	Balanced non-uniform DIF	*Unbalanced non-uniform DIF*
		Gentle Slope	Steep Slope	
**Number of items in the scale**	{4, 8}	{8}	{8}	***{8}***
**Group size (groups of equal size)**	{100, 200}	{200}	{200}	***{200}***
**Magnitude of the mean latent trait difference between the two groups**	{0, 0.1, 0.2, ***0*.*5***}	{0.1}	{0.1}	***{0*.*1}***
**DIF effect-size**	Δ = {0.25, 0.5, 1}	Δ = {0.25, 0.5, 1}*	Δ = {0.25, 0.5, 1}	**
**Proportion of items affected by DIF in the scale**	{25%, 50%, 75%}			
**Number of item response categories**	{5}			
**Item location parameters**	Percentiles of the standardized normal distribution			
**Position of the item parameter along the latent trait continuum for items affected by DIF**	Items concerned by DIF were:			
	• “Unif”: uniformly distributed along the continuum			
	• “Mean”: close to the mean of the item difficulties in the scale			
	• “Extreme”: the most difficult and easiest items of the scale			
	• “High”: the most difficult items of the scale			
	• “Low”: the easiest items of the scale			

In bold and italics, values added for the scenarios added in the current work

**Fig 2:**
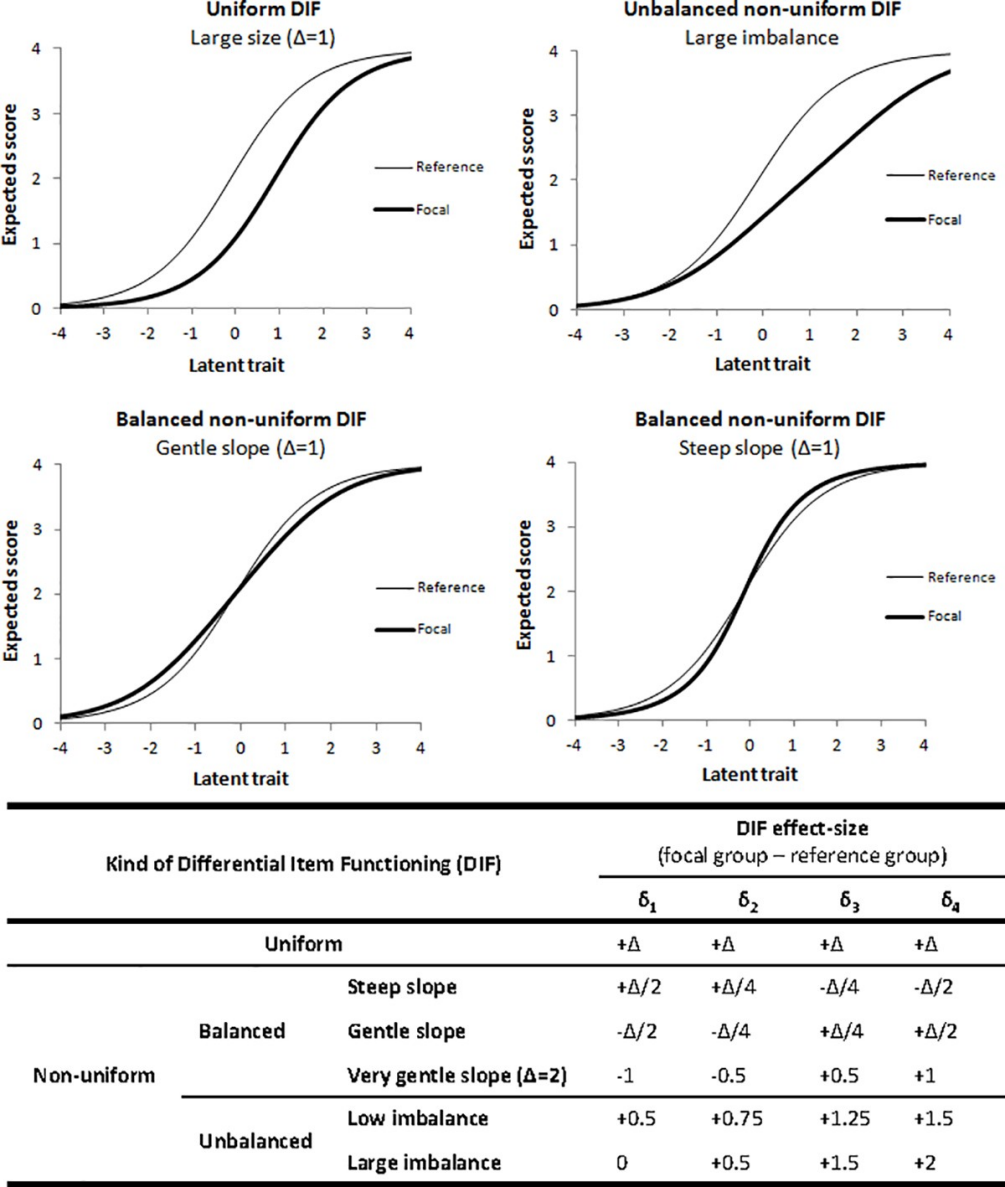
Item characteristic curves in the reference and focal groups and Differential Item Functioning (DIF) effect-size used in the simulation model for the four kinds of DIF. δ_i_: item location parameter for the item category i, Δ = {0.25, 0.5, 1}, the latent trait scale on the X-axis is given in logits.

* ***Very gentle slope*** as defined in Fig 2 was added in the current work

** ***small and large imbalance*** as defined in the Fig 2 were added in the current work

### 3.2. Methods

Datasets were generated using a partial credit model, a model for polytomous data that shares the statistical properties of the Rasch model and defines k item location parameters for items with k+1 response categories [23]. Like binary items, DIF effect-size is defined as the group difference in the item location parameter. Thus, in this model, the DIF size may vary from one category to another within the same item and thereby cause non-uniform DIF; DIF is uniform if and only if its size is identical across all the categories of the same item.

Factor values conforming to the conditions encountered in health research studies were selected (Table 2): small numbers of items in scales (4 or 8 items) with five response categories, small group size (100 or 200 with groups of equal size), and a magnitude of the mean latent trait difference between the two groups of 0 (no difference) to 0.5 standard deviation (Gaussian distribution of the simulated latent trait in both groups, with a variance equal to 1). The three factors related to the presence of DIF in the scale were: DIF effect size, proportion of items in the scale affected by DIF (25%, 50% or 75%) and the position of the item parameter along the latent trait continuum for items affected by DIF (five different distributions along the latent trait continuum). The model used to generate data is described elsewhere [19].

Four kinds of DIF were simulated using the DIF effect-size factor (Fig 2):

- Uniform DIF: the relation between the group and the response to the item is identical at every level of the latent trait, i.e. item characteristics curves for the two groups are parallel (upper-left corner of Fig 2). To generate data with uniform DIF, the DIF affecting the four item location parameters within the same item was set to be of equal size. Three sizes of uniform DIF were studied: 0.25, 0.5 and 1 logit and the item was more difficult in the focal group.
- Balanced non-uniform DIF: the relation between the group and the response depends on the level of the latent trait but the overall item difficulty is identical in the two groups. To generate data with balanced non-uniform DIF, the DIF was set to be unequal for the four item location parameters but the sum of the DIF sizes was zero, such that the item characteristic curves crossed at the latent trait level equal to zero (bottom of Fig 2). Three sizes were initially studied for both kinds of balanced non-uniform DIF, i.e. gentle slope (the slope of the item characteristic curve was gentler in the focal group) and steep slope. A fourth size for the gentle slope case was then added to the analysis: very gentle slope (Fig 2).
- Balanced non-uniform DIF with steep slope: the slope of the item characteristic curve was steeper in the focal group (bottom-right corner of Fig 2)
- Balanced non-uniform DIF with gentle slope: the slope of the item characteristic curve was gentler in the focal group (bottom-left corner of Fig 2)
- We also studied unbalanced non-uniform DIF in which the overall item difficulty in the focal group was higher than in the reference group as the sum of the DIF sizes affecting the four location parameters was positive. Two sizes of unbalanced non-uniform DIF were used to generate data: low unbalanced and large unbalanced (overall item difficulty in the focal group higher than in the low unbalanced case, Fig 2).

A latent regression partial credit model was applied to each of the 500 datasets generated for each scenario to estimate differences between groups without taking into account the presence of DIF. The *measurement bias* due to DIF was estimated as the delta between this estimated difference and the value of the mean latent trait difference between the two groups used to generate data (the “true” difference between groups). Four multivariate models of analysis of variance (ANOVA), one for each kind of DIF studied, were used to investigate the effects on the *measurement bias* of each factor manipulated in the simulation model. However, the number of simulations studied by ANOVA was huge, so a benchmark was set to distinguish between negligible and non-negligible statistically significant factor effects. To do this, the same analyses were performed on the 500 datasets generated for each of the 12 scenarios resulting from the combination of the three factors not related to DIF (number of items in the scale, group size and a magnitude of the mean latent trait difference between the two groups), with all factors related to DIF set to zero; the resulting delta between the estimated and the “true” difference between the two groups was termed *random error* instead of *measurement bias* as there was no source of DIF-related bias in the simulation model. The maximum of the mean *random error* over these 12 scenarios was used as the benchmark.

The effect of the three factors not related to DIF was studied in the case of uniform DIF and was found to be negligible. To facilitate the simulation study, it was decided to investigate each kind of non-uniform DIF with the number of items in the scale set at 8, the group size set at 200 and the magnitude of the mean latent trait difference between the two groups set at 0.1 logit (Table 2). Larger mean latent trait differences between the two groups were investigated because values studied in the previous work were small (0, 0.1 and 0.2). The minimal clinically important difference in a measurement scale is in some cases suggested to be half a standard deviation of the score, so we investigated the effect of this factor being 0.5 [24].The effect of the three DIF-related factors and their interactions were studied in each kind of DIF. Stata software was used for data generation and statistical analyses [25].

### 3.3. Results

In simulations without DIF, mean *random error* over the 500 datasets generated for each of the 12 combinations was never higher than 0.013 logit and was not affected by any of the three factors manipulated. In the following analyses, a factor was considered to have a non-negligible effect on *measurement bias* if its estimated coefficient was statistically significant and higher than 0.015 logit; it was considered to have a major effect if its estimated coefficient was higher than 0.1 logit, i.e. half of a small effect-size as defined by Cohen [26].

Although the estimated ANOVA coefficients of the effects of the three factors not related to DIF (number of items in the scale, group size and a magnitude of the mean latent trait difference between the two groups) were significant (p<0.001) they were lower than 0.015 logit in the case of uniform DIF; they were thus considered to be negligible. When the mean latent trait difference between the two groups was set at 0.5 logit, the associated estimated coefficient was significant (p<0.001) but at -0.022 logit (standard error = 0.001) thus <0.1 logit: its effect was therefore classified as small.

Multivariate ANOVA results for the three factors related to DIF for each kind of DIF are shown in Table 3. For uniform DIF, DIF effect-size, proportion of items in the scale affected by DIF and particularly the interaction between these two factors all had major effects. The effect of the position of the item parameter along the latent trait continuum for items affected by DIF on *measurement bias* was significant but not large. No DIF-related factor was found to be associated with *measurement bias* in either case of balanced non-uniform DIF. For unbalanced non-uniform DIF, the DIF effect-size and its interaction with the proportion factor had large effects on *measurement bias*, as for uniform DIF. The position factor was found to be associated but its effect was not large.

**Table 3:** Results of the multivariate analysis of variance of the measurement bias for the four kinds of Differential Item Functioning (DIF) studied.

Factor	Uniform DIF			Balanced non-uniform DIF Steep slope			Balanced non-uniform DIF Gentle slope			Unbalanced non-uniform DIF		
	Category	Estimate (SE)	*p*-value	Category	Estimate (SE)	*p*-value	Category	Estimate (SE)	*p*-value	Category	Estimate (SE)	*p*-value
**DIF effect-size**	**Δ = 0.25**	Reference	<0.001	**Δ = 0.25**	Reference	0.808	**Δ = 0.25**	Reference	0.270	**Balanced (Δ = 1)**	Reference	<0.001
	**Δ = 0.5**	0.051 (0.002)		**Δ = 0.5**	-0.001 (0.002)		**Δ = 0.5**	0.000 (0.002)		**Small imbalance**	0.204 (0.004)	
	**Δ = 1**	0.145 (0.002)		**Δ = 1**	-0.000 (0.002)		**Δ = 1**	0.002 (0.002)		**Large imbalance**	0.185 (0.004)	
							**Δ = 2 (very gentle)**	-0.003 (0.002)				
**Proportion**	**25%**	Reference	<0.001	**25%**	Reference	0.364	**25%**	Reference	0.030	**25%**	Reference	<0.001
	**50%**	0.063 (0.002)		**50%**	0.003 (0.002)		**50%**	-0.003 (0.002)		**50%**	0.000 (0.004)	
	**75%**	0.128 (0.002)		**75%**	0.002 (0.002)		**75%**	0.002 (0.002)		**75%**	0.006 (0.004)	
**Position**	**Unif**	Reference	<0.001	**Unif**	Reference	<0.001	**Unif**	Reference	<0.001	**Unif**	Reference	<0.001
	**Mean**	-0.024 (0.001)		**Mean**	0.001 (0.003)		**Mean**	0.000 (0.003)		**Mean**	-0.026 (0.003)	
	**Extreme**	-0.052 (0.001)		**Extreme**	0.001 (0.003)		**Extreme**	0.000 (0.003)		**Extreme**	-0.046 (0.003)	
	**High**	-0.037 (0.001)		**High**	0.017 (0.003)		**High**	-0.025 (0.003)		**High**	-0.084 (0.003)	
	**Low**	-0.011 (0.001)		**Low**	-0.015 (0.003)		**Low**	0.028 (0.003)		**Low**	0.037 (0.003)	
Interactioneffect-size * proportion	0.5*50%	0.064 (0.003)	<0.001							Small imbalance*50%	0.214 (0.005)	<0.001
	0.5*75%	0.129 (0.003)								**Small imbalance** *75%	0.446 (0.005)	
	1*50%	0.166 (0.003)								Large imbalance*50%	0.184 (0.005)	
	1*75%	0.366 (0.003)								**Large imbalance** *75%	0.410 (0.005)	
**Constant**		0.089 (0.002)	<0.001		-0.004 (0.003)	0.225		-0.003 (0.003)	0.312		0.017 (0.003)	<0.001

Note: Only the interaction terms which were statistically significant and with estimates higher than 0.1 were kept in the models. SE: Standard Error, Δ: Magnitude of the group difference in the location parameter; Proportion: proportion of items with DIF in the scale; Position: position of the location parameters of items with DIF along the latent trait; Unif: uniformly distributed; Mean: around the mean of the item difficulties; Extreme: high and low difficulties; High: high difficulties; Low: low difficulties

*: interaction

To summarize, the three factors not related to DIF (number of items in the scale, group size and magnitude of the mean latent trait difference between groups) were found to have negligible effects on *measurement bias*. Also, in both cases of balanced non-uniform DIF, every factor studied had a negligible effect. However, when DIF was uniform or unbalanced non-uniform, DIF effect-size, proportion of items in the scale affected by DIF and particularly the interaction between these two factors all had major effects on *measurement bias*.

## 4. Recommendations reviewed by leading experts

### 4.1. Objective and target users

We found that there is an issue with how to handle statistically significant DIF in the Rasch framework in composite measurement scales, as used in health research studies. We therefore developed recommendations for methodologists and clinicians involved in the design of new composite measurement scales within this framework or using it in their health-related studies.

### 4.2. Methods

The first draft of the recommendations was established by AR, JBH, AV and JC (biostatisticians expert in psychometrics methods: AR, JBH, JC, and epidemiologists using composite measurement scales in research: AR, JBH, AV, JC or clinical practice: JC) using information from the literature review and the simulation study. In preparation for the review, the Appraisal of Guidelines REsearch Evaluation (AGREE) instrument, not intended for non-clinical context, was adapted to the evaluation of these recommendations (S2 Table) [27]. VS pretested this instrument adapted in performing a review of the first draft of the guidelines. Then, after extensive discussion between AR, JBH, JC and VS, an informal consensus led to a second draft. An invitation to review these recommendations was then sent to 22 experts of the Rasch framework or experts in epidemiology and familiar with DIF assessment using Rasch framework models (10 in Europe, 10 in the United-States, 1 in Asia and 1 in Australia). A draft of this manuscript with a reprint of the previously published simulation study and the AGREE instrument adapted were sent to the 7 experts who accepted the invitation (list in acknowledgments section).

### 4.3. Results

Nearly 70% of the 285 articles reviewed in which the IRT framework was used involved the Rasch framework. It was the most used in “quality of life” and “medical disciplines” research fields. These recommendations related to DIF assessment in composite measurement scales using Rasch framework models are therefore relevant to most medical epidemiologists. Sample size and characteristics of the scales studied in these articles were close to the values used in the simulation model. The number of items per dimension (median = 9[6–15]) was nevertheless smaller in the simulation study but it was not associated with *measurement bias*. According to the information from the literature review, results from our simulation study, which allowed analysis of most situations encountered in practice, thus appear to be a suitable basis for recommendations.

**Recommendation 1**: *Distinguish between uniform*, *balanced non-uniform and unbalanced non-uniform DIF*.

Justification: In the simulation study, there were large effects of DIF effect-size, proportion of DIF items and their interaction on measurement bias in case of uniform or unbalanced non-uniform DIF. However, non-uniform DIF was assessed in less than a quarter of the published articles using the Rasch framework. While in most of software that allow estimation of Rasch model item parameters, DIF tests are accessible or may be programmed, only few of them, the most popular ones, include programs aimed at distinguishing uniform from non-uniform DIF: RUMM (used in 25 articles), WINSTEPS (used in 17 articles)[28,29].

**Recommendation 2**: *When statistically significant DIF is found*, *its effect-size should be estimated as the group difference in the item location parameter(s)*.

Justification: DIF effect size estimates were provided in only 18 (37%) of the 49 articles in which statistically significant DIF was detected within the Rasch framework. However, the simulation study revealed that this information is essential to evaluate the consequences of DIF. Indeed, ANOVA coefficients of this factor and of its interaction with the proportion of items in the scale affected by DIF were higher than 0.1 logit for both uniform and unbalanced non-uniform DIF.

**Recommendation 3**: *Due to the presence of statistically significant DIF*:

- *If the scale has been modified*, *provide information on how it has been modified*
- *If the scale has not been modified*, *provide recommendations on how to take the presence of DIF with meaningful consequences into account when using the scale in practice*, *i*.*e*. *when*:
  - Statistically significant uniform DIF is found in more than 25% of the items within a dimension
  - *Statistically significant uniform DIF is found with an effect-size higher than 0*.*25 logit*
  - Statistically significant unbalanced non-uniform DIF is found

Justification: In 31 (44%) of the 71 articles in which statistically significant DIF was found, there was no modification of the scale, and no recommendation on how to handle the presence of DIF in practice. DIF effect-size thresholds found in the simulation study were different to those encountered in most published studies; this should be underlined as the threshold can have substantial effects on measurement bias in practice [12,18].

**Recommendation 4**: *If several items exhibit statistically significant DIF within the dimension but in opposite direction*, *assess its effects at the dimension level empirically*.

Justification: If the effects from items exhibiting DIF for one group are cancelled by those of other items that exhibit DIF for another group in the dimension, this is called the “DIF cancellation” phenomenon [30–33]. This was not addressed in the simulation study as it involved too many situations to perform. However, a kind of “cancellation” phenomenon was observed at the item level for balanced non-uniform DIF in which DIF-related factors had negligible effects. This was not observed for unbalanced non-uniform DIF. Therefore, the effects from items exhibiting DIF for one group may not be cancelled by those of other items that exhibit DIF for another group if the sum of the effect-sizes of the DIF exhibited by all the items within the dimension is not zero (unbalanced).

## 5. Strengths and limits

Following the review, recommendations were not modified but their justification was improved and their application field more precisely defined. Median of reviewers’ ratings to each AGREE item and scores to the six AGREE domains are reported in the S2 Table. Scores were higher than 70% in every domain except the “Applicability domain”. As other improvements, a box (S1 Box) gathering the recommendations altogether was supplied and strengths and limits of each method used in this work clarified.

One of the major strengths of these recommendations is that they are based on results from a simulation study closely linked to their field of application, i.e. health research studies in which the Rasch framework is used, as revealed by the literature review. However, in more than half of the articles reviewed, measurement invariance was assessed using methods from the CFA framework. As perspective, similar work to establish recommendations for the CFA framework would therefore be valuable, as well as for 2PL IRT models.

We propose only four recommendations to facilitate their application, and recommendation 3 provides practical criteria to identify statistically significant DIF as not meaningful in many situations. Applying these recommendations will thus reduce resources as, in these situations, it will reduce the need for statisticians with expertise with models for taking DIF into account. Finally, using a threshold of 0.1 logit (not completely arbitrary as equal to half a small effect-size as defined by Cohen), there was no effect sufficiently large to be relevant in practice found in the simulation study concerning the position of the item affected by DIF along the latent trait continuum. No recommendation was thus formulated on this factor but future updates of these recommendations (within 10 years) will be performed based on scenarios which were not considered in our simulation study. It will also involve literature reviews to assess their application in practice and consequences on how DIF is handled by scale users, as well as any new theoretical and methodological approaches to study DIF. Finally, although some statistical software facilitate study of DIF using the Rasch model (RUMM, Stata, R), their use to characterize DIF as meaningful or not can be complicated and thus impede the application of these recommendations [28,34,35]. To raise these barriers to the application of the recommendations, development of modules or packages for existing statistics software is being considered.

This study has some limitations that highlight the need for further works on detection and characterization of statistically significant DIF. Indeed, these recommendations do not concern best methods for DIF detection and, as revealed in the literature study, these methods are numerous, so researchers should be interested in some guidance in this area. For example, guidance for assessing DIF iteratively to distinguish between real and artificial DIF has been published in the last decade and this is an imperative step before applying the present recommendations [36,37]. Also, the literature review and the simulation study were designed in order to focus on health research studies, however, this is not the only research domain in which DIF is a subject of concern and, to our knowledge, such recommendations do not exist in any domain. Incidentally, further work should also focus on recommendations on the next step, i.e. on best practice when DIF is identified and found statistically significant and relevant. Finally, information on the best methods to take meaningful DIF into account is scarce in the literature and this will be further studied to enrich these first recommendations on DIF consideration in health research studies. In this respect, it is to note that in some cases, it can be quite difficult to decide on whether or not applying a method to take meaningful DIF into account as it may reduce the validity of the assessment if the source of the DIF is conceptually relevant to the attribute being measured [38].

## 6. Conclusions

Our combination of a literature review and a simulation study led to the first consensual recommendations reviewed by international experts for the assessment of DIF within the Rasch framework in short composite measurement scales. There is a growing interest in DIF in health research, and these recommendations will help researchers conducting DIF assessment studies. They will also help scale users because the information provided in DIF assessment studies will be clearer on how to report and handle DIF into account in practice. This will contribute to better accuracy of results in epidemiological studies by improving the measurement properties of composite measurement scales, increasingly used in health research.

## Supporting information

S1 Table Evaluation of the effects of the presence of DIF and practices and/or recommendation produced for its management in practice. N = 71 articles in which statistically significant DIF was found.
(DOCX)

S2 Table Median of the rating (from 1-“strongly disagree” to 7-“strongly agree”) by the seven reviewers to each items of the Appraisal of Guidelines for Research & Evaluation II (AGREE II) adapted for the evaluation of methodological recommendations and score to each of the six domains. (DOCX)

S1 List List of the references of the 285 articles included in the literature review. References with 2015 as year of publication were published online during the search period: November 11, 2013 and November 10, 2014.
(DOCX)

S1 Box Median of the rating (from 1-“strongly disagree” to 7-“strongly agree”) by the seven reviewers to each items of the Appraisal of Guidelines for Research & Evaluation II (AGREE II) adapted for the evaluation of methodological recommendations and score to each of the six domains. (DOCX)
