# Supplementary material for: Differential Item Functioning (DIF) in composite health measurement scale: Recommendations for characterizing DIF with meaningful consequences within the Rasch model framework
Source: PLoS One. 2019 Apr 9;14(4):e0215073. doi: 10.1371/journal.pone.0215073 (PMC6456214; doi:10.1371/journal.pone.0215073)
Supplement: S1 Table — N = 71 articles in which statistically significant DIF was found. (DOCX) [file pone.0215073.s001.docx]

**S1 Table:** Evaluation of the effects of the presence of DIF and practices and/or recommendation produced for its management in practice.

(n=71 articles in which statistically significant DIF was found)

| **Empirical evaluation of the Impact of the presence of DIF (n=18)** | |
| --- | --- |
| **On the latent trait estimates** **(n=6)**: latent trait estimated without consideration of the presence of DIF was compared to  → Latent trait estimated using the modified scale, i.e. without the items exhibiting DIF  → Latent trait estimated using the original scale but with different values of parameters in each group for items exhibiting DIF  The impact was considered as negligible if :   - Difference between mean latent trait < 0.2 - Pearson correlation or intra-class correlation coefficient > 0.8 - High proportion of subjects for whom the difference between the latent trait estimated with or without consideration of DIF < SEM - Graphically negligible on the Bland and Altman plot   **On the expected scale score (n=6)**: expected scale score computed without consideration of the presence of DIF in the scale was compared to the expected scale score using the original scale but with different values of parameters in each group for items exhibiting DIF  The impact was considered as negligible if :   - Highest difference between the expected score with or without consideration of DIF < SEM - Graphically negligible when comparing the test characteristic curves with or without consideration of DIF   **On the results of statistical analyses (n=5)**: statistical analyses were performed using the latent trait estimated (or the score computed) without consideration of DIF and using the latent trait estimated (or the score computed) using the original scale but with different values of parameters in each group for items exhibiting DIF  The impact was considered as negligible if:   - Same conclusions were drawn from the results of the statistical analyses   **On item and person reliabilities (n=1)**: item and person reliabilities were computed with or without items exhibiting DIF  The impact was considered as negligible if:   - Reliabilities computed without items exhibiting DIF >0.8 | |
| **Practices and/or recommendations produced by the authors on how to handle the presence of DIF in practice (N=71)** | |
| **No modification of the scale or of its use in practice (n=45)**   - The consequence of the presence of DIF empirically evaluated and considered to be negligible (n=14), - No reason or too few items affected by DIF, or DIF cancellation phenomenon (n=10) - Further investigations recommended (n=21) | **Modification(s) of the scale or of its use in practice (n=26)**   - Recommendation to split items exhibiting DIF to calibrate them separately in each group when the scale is used in a study (n=12) - Remove items exhibiting DIF from the scale (n=11) - Reformulate items exhibiting DIF (n=2) - Combine items exhibiting DIF with other items of the scale (n=3) |
