# Supplementary material for: Differential Item Functioning (DIF) in composite health measurement scale: Recommendations for characterizing DIF with meaningful consequences within the Rasch model framework
Source: PLoS One. 2019 Apr 9;14(4):e0215073. doi: 10.1371/journal.pone.0215073 (PMC6456214; doi:10.1371/journal.pone.0215073)
Supplement: S2 Table — (DOCX) [file pone.0215073.s002.docx]

**S2 -Table**: Median of the rating (from 1-“strongly disagree” to 7-“strongly agree”) by the seven reviewers to each items of the Appraisal of Guidelines for Research & Evaluation II (AGREE II) adapted for the evaluation of methodological recommendations and score to each of the six domains

| **Items of the AGREE II modified** | **Median** | **Domain** | **Score*** |
| --- | --- | --- | --- |
| **1 -** The overall objective(s) of the guideline is (are) specifically described. | 7 | **Scope and purpose:** Concerned with overall aim of the guideline, specific methodological questions and target material on which the recommendations will apply. | 90% |
| **2 -** The methodological question(s) covered by the guideline is (are) specifically described. | 7 |  |  |
| **3 -** The material (study design, statistical method, etc.) to which the guideline is meant to apply is specifically described. | 7 |  |  |
| **4 -** The guideline development group includes individuals from all relevant professional groups. | 5 | **Stakeholder Involvement:** Focusing on the extent to which the guideline was developed by the appropriate stakeholders and represents the views of its intended users. | 71% |
| **5 -** The target users of the guideline are clearly defined. | 6 |  |  |
| **6 -** Appropriate methods were used to search for evidence. | 5 | **Rigour of Development**: Related to the process used to gather and synthesize the evidence, the methods to formulate the recommendations, and to update them | 73% |
| **7 -** Methods used to search for evidence are clearly described. | 6 |  |  |
| **8 -** The strengths and limitations of the methods used to search for evidence are clearly described. | 5 |  |  |
| **9 -** The methods for formulating the recommendations are clearly described. | 5 |  |  |
| **10 -** There is an explicit link between the recommendations and the supporting evidence. | 6 |  |  |
| **11 -** The guideline has been externally reviewed by experts prior to its publication. | 6,5 |  |  |
| **12 -** A procedure for updating the guideline is provided. | 6 |  |  |
| **13 -** The recommendations are specific and unambiguous | 5 | **Clarity of Presentation:**Deals with language, structure, and format of the guideline | 71% |
| **14 -** Key recommendations are easily identifiable. | 6 |  |  |
| **15 -** The guideline describes facilitators and barriers to its application. | 4 | **Applicability:** Pertaining to the likely barriers and facilitators to implementation,  strategies to improve uptake, and resource implications of applying the guideline. | 56% |
| **16 -** The guideline provides advice and/or tools on how the recommendations can be put into practice. | 5 |  |  |
| **17 -** The potential resource implications of applying the recommendations have been considered. | 3 |  |  |
| **18 -** The guideline presents monitoring and/or auditing criteria. | 3 |  |  |
| **19 -** The views of the funding body have not influenced the content of the guideline. | 7 | **Editorial Independence:** Concerned with the formulation of recommendations not being unduly biased with competing interests | 98% |
| **20 -** Competing interests of guideline development group members have been recorded and addressed. | 7 |  |  |
| **OVERALL 1**: Rate the overall quality of this guideline | 5 |  |  |
| **OVERALL 2**: I would recommend this guideline for use | **Yes** (n=1), **Yes with modifications** (n=5), **No** (n=1) | | |

*computed as the sum of all the scores of the items in a domain, scaled as a percentage of the maximum possible score for that domain

In grey, item removed as suggested by reviewers as not appropriate for the evaluation of methodological recommendations
