# Supplementary material for: Differential Item Functioning (DIF) in composite health measurement scale: Recommendations for characterizing DIF with meaningful consequences within the Rasch model framework
Source: PLoS One. 2019 Apr 9;14(4):e0215073. doi: 10.1371/journal.pone.0215073 (PMC6456214; doi:10.1371/journal.pone.0215073)
Supplement: S1 List — References with 2015 as year of publication were published online during the search period: November 11, 2013 and November 10, 2014. (DOCX) [file pone.0215073.s003.docx]

**S1 List of the references of the 285 articles included in the literature review**

(References with 2015 as year of publication were published online during the search period: November 11, 2013 and November 10, 2014)

Aati, O., Taylor, W.J., Siegert, R.J., Horne, A., House, M.E., Tan, P., Drake, J., Stamp, L.K., Dalbeth, N., 2014. Development of a patient-reported outcome measure of tophus burden: the Tophus Impact Questionnaire (TIQ-20). Ann. Rheum. Dis. 74(12):2144-50. doi:10.1136/annrheumdis-2014-205671

Ackerman, I.N., Busija, L., Tacey, M.A., Bohensky, M.A., Ademi, Z., Brand, C.A., Liew, D., 2014. Performance of the assessment of quality of life measure in people with hip and knee joint disease and implications for research and clinical use. Arthritis Care Res (Hoboken) 66, 481–488. doi:10.1002/acr.22129

Aggarwal, A.N., Agarwal, R., Gupta, D., 2014. Abbreviated World Health Organization Quality of Life questionnaire (WHOQOL-Bref) in north Indian patients with bronchial asthma: an evaluation using Rasch analysis. NPJ Prim Care Respir Med 24, 14001. doi:10.1038/npjpcrm.2014.1

Aiena, B.J., Baczwaski, B.J., Schulenberg, S.E., Buchanan, E.M., 2015. Measuring resilience with the RS-14: a tale of two samples. J Pers Assess 97, 291–300. doi:10.1080/00223891.2014.951445

Alessandri, G., Fagnani, C., Di Gennaro, G., Meldolesi, G.N., Pasquini, P., Caroppo, E., Martinotti, G., Toni, A., Fabi, E., Picardi, A., 2014a. Measurement invariance of the experiences in close relationships questionnaire across different populations. Span J Psychol 17, E22. doi:10.1017/sjp.2014.19

Alessandri, G., Vecchione, M., Donnellan, B.M., Eisenberg, N., Caprara, G.V., Cieciuch, J., 2014b. On the cross-cultural replicability of the resilient, undercontrolled, and overcontrolled personality types. J Pers 82, 340–353. doi:10.1111/jopy.12065

Andersen, K.G., Christensen, K.B., Kehlet, H., Bidstup, P.E., 2014. The Effect of Pain on Physical Functioning after Breast Cancer Treatment: Development and Validation of an Assessment Tool. Clin J Pain. 31, 794-802. doi:10.1097/AJP.0000000000000156

Andrae, D.A., Covington, P.S., Patrick, D.L., 2014. Item-level assessment of the irritable bowel syndrome quality of life questionnaire in patients with diarrheal irritable bowel syndrome. Clin Ther 36, 663–679. doi:10.1016/j.clinthera.2014.04.009

Årestedt, K., Ågren, S., Flemme, I., Moser, D.K., Strömberg, A., 2015. A psychometric evaluation of the four-item version of the Control Attitudes Scale for patients with cardiac disease and their partners. Eur J Cardiovasc Nurs 14, 317–325. doi:10.1177/1474515114529685

Ashley, L., Smith, A.B., Jones, H., Velikova, G., Wright, P., 2014. Traditional and Rasch psychometric analyses of the Quality of Life in Adult Cancer Survivors (QLACS) questionnaire in shorter-term cancer survivors 15 months post-diagnosis. J Psychosom Res 77, 322–329. doi:10.1016/j.jpsychores.2014.07.007

Ashley, L., Smith, A.B., Keding, A., Jones, H., Velikova, G., Wright, P., 2013. Psychometric evaluation of the revised Illness Perception Questionnaire (IPQ-R) in cancer patients: confirmatory factor analysis and Rasch analysis. J Psychosom Res 75, 556–562. doi:10.1016/j.jpsychores.2013.08.005

Ayalon, L., 2015. Reports of Elder Neglect by Older Adults, Their Family Caregivers, and Their Home Care Workers: A Test of Measurement Invariance. J Gerontol B Psychol Sci Soc Sci 70, 432–442. doi:10.1093/geronb/gbu051

Ayele, D.G., Zewotir, T., Mwambi, H., 2014. Using Rasch modeling to re-evaluate rapid malaria diagnosis test analyses. Int J Environ Res Public Health 11, 6681–6691. doi:10.3390/ijerph110706681

Bagby, R.M., Ayearst, L.E., Morariu, R.A., Watters, C., Taylor, G.J., 2014. The internet administration version of the 20-item Toronto alexithymia scale. Psychol Assess 26, 16–22. doi:10.1037/a0034316

Ballert, C.S., Post, M.W., Brinkhof, M.W., Reinhardt, J.D., SwiSCI Study Group, 2015. Psychometric properties of the Nottwil Environmental Factors Inventory Short Form. Arch Phys Med Rehabil 96, 233–240. doi:10.1016/j.apmr.2014.09.004

Ballert, C.S., Stucki, G., Biering-Sørensen, F., Cieza, A., 2014. Towards the development of clinical measures for spinal cord injury based on the International Classification of Functioning, Disability and Health with Rasch analyses. Arch Phys Med Rehabil 95, 1685–1694. doi:10.1016/j.apmr.2014.05.006

Balsamo, M., Giampaglia, G., Saggino, A., 2014. Building a new Rasch-based self-report inventory of depression. Neuropsychiatr Dis Treat 10, 153–165. doi:10.2147/NDT.S53425

Barbic, S.P., Durisko, Z., Andrews, P.W., 2014. Measuring the bright side of being blue: a new tool for assessing analytical rumination in depression. PLoS ONE 9, e112077. doi:10.1371/journal.pone.0112077

Barbosa-Leiker, C., McPherson, S., Mamey, M.R., Burns, G.L., Roll, J., 2014a. Psychometric properties of the adjective rating scale for withdrawal across treatment groups, gender, and over time. J Subst Abuse Treat 46, 251–256. doi:10.1016/j.jsat.2013.08.020

Barbosa-Leiker, C., Strand, P.S., Mamey, M.R., Downs, A., 2014b. Psychometric properties of the Emotion Understanding Assessment with Spanish- and English-speaking preschoolers attending Head Start. Assessment 21, 628–636. doi:10.1177/1073191114524017

Barbot, B., Crossman, E., Hunter, S.R., Grigorenko, E.L., Luthar, S.S., 2014a. Reciprocal influences between maternal parenting and child adjustment in a high-risk population: a 5-year cross-lagged analysis of bidirectional effects. Am J Orthopsychiatry 84, 567–580. doi:10.1037/ort0000012

Barbot, B., Hein, S., Luthar, S.S., Grigorenko, E.L., 2014b. Capturing Age-group Differences and Developmental Change with the BASC Parent Rating Scales. J Appl Dev Psychol 35, 294–303. doi:10.1016/j.appdev.2014.04.003

Baumert, A., Beierlein, C., Schmitt, M., Kemper, C.J., Kovaleva, A., Liebig, S., Rammstedt, B., 2014. Measuring four perspectives of justice sensitivity with two items each. J Pers Assess 96, 380–390. doi:10.1080/00223891.2013.836526

Baylor, C., McAuliffe, M.J., Hughes, L.E., Yorkston, K., Anderson, T., Kim, J., Amtmann, D., 2014. A differential item functioning (DIF) analysis of the Communicative Participation Item Bank (CPIB): comparing individuals with Parkinson’s disease from the United States and New Zealand. J. Speech Lang. Hear. Res. 57, 90–95. doi:10.1044/1092-4388(2013/12-0414)

Belon, K.E., McLaughlin, E.A., Smith, J.E., Bryan, A.D., Witkiewitz, K., Lash, D.N., Winn, J.L., 2015. Testing the measurement invariance of the Eating Disorder Inventory in nonclinical samples of Hispanic and Caucasian women. Int J Eat Disord 48, 262–270. doi:10.1002/eat.22286

Benedetti, M.G., Franchignoni, F., Morri, M., Franchini, N., Natali, E., Giordano, A., 2014. Rasch analysis of the Iowa Level of Assistance Scale in patients with total hip and knee arthroplasty. Int J Rehabil Res 37, 118–124. doi:10.1097/MRR.0000000000000043

Bentler, S.E., Morgan, R.O., Virnig, B.A., Wolinsky, F.D., 2014. Evaluation of a patient-reported continuity of care model for older adults. Qual Life Res 23, 185–193. doi:10.1007/s11136-013-0472-z

Bentley, K.H., Gallagher, M.W., Carl, J.R., Barlow, D.H., 2014. Development and validation of the Overall Depression Severity and Impairment Scale. Psychol Assess 26, 815–830. doi:10.1037/a0036216

Borsa, J.C., Damásio, B.F., Bandeira, D.R., Gremigni, P., 2013. The Peer Aggressive and Reactive Behavior Questionnaire (PARB-Q): measurement invariance across Italian and Brazilian children, gender and age. Child Psychiatry Hum Dev 44, 766–776. doi:10.1007/s10578-013-0368-8

Bradshaw, C.P., Waasdorp, T.E., Debnam, K.J., Johnson, S.L., 2014. Measuring school climate in high schools: a focus on safety, engagement, and the environment. J Sch Health 84, 593–604. doi:10.1111/josh.12186

Brewster, P.W.H., Tuokko, H., MacDonald, S.W.S., 2014. Measurement equivalence of neuropsychological tests across education levels in older adults. J Clin Exp Neuropsychol 36, 1042–1054. doi:10.1080/13803395.2014.967661

Brothers, A., Chui, H., Diehl, M., 2014. Measuring future time perspective across adulthood: development and evaluation of a brief multidimensional questionnaire. Gerontologist 54, 1075–1088. doi:10.1093/geront/gnu076

Brown, S.D., Unger Hu, K.A., Mevi, A.A., Hedderson, M.M., Shan, J., Quesenberry, C.P., Ferrara, A., 2014. The multigroup ethnic identity measure-revised: measurement invariance across racial and ethnic groups. J Couns Psychol 61, 154–161. doi:10.1037/a0034749

Brummelman, E., Thomaes, S., Nelemans, S.A., Orobio de Castro, B., Bushman, B.J., 2015. My child is God’s gift to humanity: development and validation of the Parental Overvaluation Scale (POS). J Pers Soc Psychol 108, 665–679. doi:10.1037/pspp0000012

Brunet, J., Sabiston, C.M., Chaiton, M., Low, N.C.P., Contreras, G., Barnett, T.A., O’Loughlin, J.L., 2014. Measurement invariance of the depressive symptoms scale during adolescence. BMC Psychiatry 14, 95. doi:10.1186/1471-244X-14-95

Bruyneel, L., Li, B., Squires, A., Spotbeen, S., Meuleman, B., Lesaffre, E., Sermeus, W., 2014. Bayesian Multilevel MIMIC Modeling for Studying Measurement Invariance in Cross-group Comparisons. Med Care. doi:10.1097/MLR.0000000000000164

Buchan, H., Sunderland, M., Carragher, N., Batterham, P., Slade, T., 2014. Investigating age-related differences in responses to screening items for internalising disorders in three national surveys. J Affect Disord 152–154, 229–236. doi:10.1016/j.jad.2013.09.015

Bujacz, A., Vittersø, J., Huta, V., Kaczmarek, L.D., 2014. Measuring hedonia and eudaimonia as motives for activities: cross-national investigation through traditional and Bayesian structural equation modeling. Front Psychol 5, 984. doi:10.3389/fpsyg.2014.00984

Buz, J., Pérez-Arechaederra, D., 2014. Psychometric properties and measurement invariance of the Spanish version of the 11-item De Jong Gierveld loneliness scale. Int Psychogeriatr 26, 1–12. doi:10.1017/S1041610214000507

Cameron, I.M., Scott, N.W., Adler, M., Reid, I.C., 2014. A comparison of three methods of assessing differential item functioning (DIF) in the Hospital Anxiety Depression Scale: ordinal logistic regression, Rasch analysis and the Mantel chi-square procedure. Qual Life Res 23, 2883–2888. doi:10.1007/s11136-014-0719-3

Campos, J.A.D.B., Carrascosa, A.C., Zucoloto, M.L., Maroco, J., 2014. Validation of a measuring instrument for the perception of oral health in women. Braz Oral Res 28. Doi : 10.1590/1807-3107BOR-2014.vol28.0033

Cannoni, E., Di Norcia, A., Bombi, A.S., Di Giunta, L., 2015. The Bicycle Drawing Test: What Does It Measure in Developmentally Typical Children? Assessment 22, 629–639. doi:10.1177/1073191114555338

Caqueo-Urízar, A., Boyer, L., Boucekine, M., Auquier, P., 2014. Spanish cross-cultural adaptation and psychometric properties of the Schizophrenia Quality of Life short-version questionnaire (SQoL18) in 3 middle-income countries: Bolivia, Chile and Peru. Schizophr. Res. 159, 136–143. doi:10.1016/j.schres.2014.08.013

Caronni, A., Zaina, F., Negrini, S., 2014. Improving the measurement of health-related quality of life in adolescent with idiopathic scoliosis: the SRS-7, a Rasch-developed short form of the SRS-22 questionnaire. Res Dev Disabil 35, 784–799. doi:10.1016/j.ridd.2014.01.020

Carragher, N., Krueger, R.F., Eaton, N.R., Markon, K.E., Keyes, K.M., Blanco, C., Saha, T.D., Hasin, D.S., 2014. ADHD and the externalizing spectrum: direct comparison of categorical, continuous, and hybrid models of liability in a nationally representative sample. Soc Psychiatry Psychiatr Epidemiol 49, 1307–1317. doi:10.1007/s00127-013-0770-3

Cham, H., Hughes, J.N., West, S.G., Im, M.H., 2014. Assessment of adolescents’ motivation for educational attainment. Psychol Assess 26, 642–659. doi:10.1037/a0036213

Chang, Y.-J., Chang, C.-H., Peng, C.-L., Wu, H.-C., Lin, H.-C., Wang, J.-Y., Li, T.-C., Yeh, Y.-C., Liang, W.-M., 2014. Measurement equivalence and feasibility of the EORTC QLQ-PR25: paper-and-pencil versus touch-screen administration. Health Qual Life Outcomes 12, 23. doi:10.1186/1477-7525-12-23

Chapman, B., Fiscella, K., Duberstein, P., Kawachi, I., Muennig, P., 2014. Measurement confounding affects the extent to which verbal IQ explains social gradients in mortality. J Epidemiol Community Health 68, 728–733. doi:10.1136/jech-2013-203741

Cheifetz, O., Packham, T.L., Macdermid, J.C., 2014. Rasch analysis of the Edmonton Symptom Assessment System and research implications. Curr Oncol 21, e186-194. doi:10.3747/co.21.1735

Chen, H., Mui, A.C., 2014. Factorial validity of the Center for Epidemiologic Studies Depression Scale short form in older population in China. Int Psychogeriatr 26, 49–57. doi:10.1017/S1041610213001701

Chen, Y.-L., Pan, A.-W., Chung, L., Chen, T.-J., 2015. Examining the validity and reliability of the Taita symptom checklist using Rasch analysis. J. Formos. Med. Assoc. 114, 221–230. doi:10.1016/j.jfma.2013.10.004

Chiorri, C., Day, T., Malmberg, L.-E., 2014. An approximate measurement invariance approach to within-couple relationship quality. Front Psychol 5, 983. doi:10.3389/fpsyg.2014.00983

Chung, H., Kim, J., Cook, K.F., Askew, R.L., Revicki, D.A., Amtmann, D., 2014. Testing measurement invariance of the patient-reported outcomes measurement information system pain behaviors score between the US general population sample and a sample of individuals with chronic pain. Qual Life Res 23, 239–244. doi:10.1007/s11136-013-0463-0

Cooper-Vince, C.E., Emmert-Aronson, B.O., Pincus, D.B., Comer, J.S., 2014. The diagnostic utility of separation anxiety disorder symptoms: an item response theory analysis. J Abnorm Child Psychol 42, 417–428. doi:10.1007/s10802-013-9788-y

Costa, D.S.J., Aaronson, N.K., Fayers, P.M., Pallant, J.F., Velikova, G., King, M.T., 2015. Testing the measurement invariance of the EORTC QLQ-C30 across primary cancer sites using multi-group confirmatory factor analysis. Qual Life Res 24, 125–133. doi:10.1007/s11136-014-0799-0

Coste, J., Tissier, F., Pouchot, J., Ecosse, E., Rouquette, A., Bertagna, X., Libé, R., Viallon, V., 2014. Rasch analysis for assessing unidimensionality and identifying measurement biases of malignancy scores in oncology. The example of the Weiss histopathological system for the diagnosis of adrenocortical cancer. Cancer Epidemiol 38, 200–208. doi:10.1016/j.canep.2014.02.004

Court, H., Forty, L., Jones, L., Gordon-Smith, K., Jones, I., Craddock, N., Smith, D.J., 2014. Improving the psychometric utility of the hypomania checklist (HCL-32): a Rasch analysis approach. J Affect Disord 152–154, 448–453. doi:10.1016/j.jad.2013.10.014

Cox, S.D., Pakenham, K.I., 2014. Confirmatory factor analysis and invariance testing of the Young Carer of Parents Inventory (YCOPI). Rehabil Psychol 59, 439–452. doi:10.1037/a0035860

Crome, E., Baillie, A., 2015. Social anxiety disorder diagnostic criteria perform equally across age, comorbid diagnosis, and performance/interaction subtypes. Anxiety Stress Coping 28, 179–191. doi:10.1080/10615806.2014.930445

da Silva, W.R., Dias, J.C.R., Maroco, J., Campos, J.A.D.B., 2014. Confirmatory factor analysis of different versions of the Body Shape Questionnaire applied to Brazilian university students. Body Image 11, 384–390. doi:10.1016/j.bodyim.2014.06.001

Dakanalis, A., Zanetti, M.A., Clerici, M., Madeddu, F., Riva, G., Caccialanza, R., 2013. Italian version of the Dutch Eating Behavior Questionnaire. Psychometric proprieties and measurement invariance across sex, BMI-status and age. Appetite 71, 187–195. doi:10.1016/j.appet.2013.08.010

Darzins, S., Imms, C., Di Stefano, M., Taylor, N.F., Pallant, J.F., 2014. Evaluation of the internal construct validity of the Personal Care Participation Assessment and Resource Tool (PC-PART) using Rasch analysis. BMC Health Serv Res 14, 543. doi:10.1186/s12913-014-0543-z

de Farias Júnior, J.C., Mendonça, G., Florindo, A.A., de Barros, M.V.G., 2014. Reliability and validity of a physical activity social support assessment scale in adolescents--ASAFA Scale. Rev Bras Epidemiol 17, 355–370. doi: 10.1590/1809-4503201400020006ENG

Delisle, V.C., Kwakkenbos, L., Hudson, M., Baron, M., Thombs, B.D., Canadian Scleroderma Research Group, 2014. An assessment of the measurement equivalence of English and French versions of the Center for Epidemiologic Studies Depression (CES-D) Scale in systemic sclerosis. PLoS ONE 9, e102897. doi:10.1371/journal.pone.0102897

Dere, J., Watters, C.A., Yu, S.C.-M., Bagby, R.M., Ryder, A.G., Harkness, K.L., 2015. Cross-cultural examination of measurement invariance of the Beck Depression Inventory-II. Psychol Assess 27, 68–81. doi:10.1037/pas0000026

Devine, J., Otto, C., Rose, M., Barthel, D., Fischer, F., Mühlan, H., Mülhan, H., Nolte, S., Schmidt, S., Ottova-Jordan, V., Ravens-Sieberer, U., 2015. A new computerized adaptive test advancing the measurement of health-related quality of life (HRQoL) in children: the Kids-CAT. Qual Life Res 24, 871–884. doi:10.1007/s11136-014-0812-7

Devy, R., Lehert, P., Varlan, E., Genty, M., Edan, G., 2015. Improving the quality of life of multiple sclerosis patients through coping strategies in routine medical practice. Neurol. Sci. 36, 85–90. doi:10.1007/s10072-014-1900-8

Di Pietro, F., Catley, M.J., McAuley, J.H., Parkitny, L., Maher, C.G., Costa, L. da C.M., Macedo, L.G., Williams, C.M., Moseley, G.L., 2014. Rasch analysis supports the use of the Pain Self-Efficacy Questionnaire. Phys Ther 94, 91–100. doi:10.2522/ptj.20130217

Dmitrieva, N.O., Fyffe, D., Mukherjee, S., Fieo, R., Zahodne, L.B., Hamilton, J., Potter, G.G., Manly, J.J., Romero, H.R., Mungas, D., Gibbons, L.E., 2015. Demographic characteristics do not decrease the utility of depressive symptoms assessments: examining the practical impact of item bias in four heterogeneous samples of older adults. Int J Geriatr Psychiatry 30, 88–96. doi:10.1002/gps.4121

Dong, L., Wu, H., Waldman, I.D., 2014. Measurement and structural invariance of the antisocial process screening device. Psychol Assess 26, 598–608. doi:10.1037/a0035139

Doron, J., Trouillet, R., Gana, K., Boiché, J., Neveu, D., Ninot, G., 2014. Examination of the hierarchical structure of the brief COPE in a French sample: empirical and theoretical convergences. J Pers Assess 96, 567–575. doi:10.1080/00223891.2014.886255

dos Santos, A.A.A., Noronha, A.P.P., Rueda, F.J.M., Segovia, J.L., 2014. Bender-gradual scoring system: performance of Brazilian and Peruvian children. Percept Mot Skills 118, 897–908. doi:10.2466/03.10.PMS.118k25w7

Duku, E., Szatmari, P., Vaillancourt, T., Georgiades, S., Thompson, A., Liu, X.-Q., Paterson, A.D., Bennett, T., 2013. Measurement equivalence of the autism symptom phenotype in children and youth. J Child Psychol Psychiatry 54, 1346–1355. doi:10.1111/jcpp.12103

Dumenci, L., Matsuyama, R., Riddle, D.L., Cartwright, L.A., Perera, R.A., Chung, H., Siminoff, L.A., 2014. Measurement of cancer health literacy and identification of patients with limited cancer health literacy. J Health Commun 19 Suppl 2, 205–224. doi:10.1080/10810730.2014.943377

Dür, M., Steiner, G., Fialka-Moser, V., Kautzky-Willer, A., Dejaco, C., Prodinger, B., Stoffer, M.A., Binder, A., Smolen, J., Stamm, T.A., 2014. Development of a new occupational balance-questionnaire: incorporating the perspectives of patients and healthy people in the design of a self-reported occupational balance outcome instrument. Health Qual Life Outcomes 12, 45. doi:10.1186/1477-7525-12-45

Edelen, M.O., Tucker, J.S., Shadel, W.G., Stucky, B.D., Cerully, J., Li, Z., Hansen, M., Cai, L., 2014. Development of the PROMIS health expectancies of smoking item banks. Nicotine Tob. Res. 16 Suppl 3, S223-231. doi:10.1093/ntr/ntu053

Elosua Oliden, P., Mujika Lizaso, J., 2014. Impact of family language and testing language on reading performance in a bilingual educational context. Psicothema 26, 328–335. doi:10.7334/psicothema2013.344

Fagnani, C., Medda, E., Stazi, M.A., Caprara, G.V., Alessandri, G., 2014. Investigation of age and gender effects on positive orientation in Italian twins. Int J Psychol 49, 453–461. doi:10.1002/ijop.12053

Falkenström, F., Hatcher, R.L., Holmqvist, R., 2015. Confirmatory Factor Analysis of the Patient Version of the Working Alliance Inventory-Short Form Revised. Assessment 22, 581–593. doi:10.1177/1073191114552472

Fang, Q., Freedenthal, S., Osman, A., 2015. Validation of the Suicide Resilience Inventory-25 with American and Chinese college students. Suicide Life Threat Behav 45, 51–64. doi:10.1111/sltb.12108

Farin, E., Schmidt, E., Gramm, L., 2014. Patient communication competence: development of a German questionnaire and correlates of competent patient behavior. Patient Educ Couns 94, 342–350. doi:10.1016/j.pec.2013.11.005

Feinn, R., Schumann, K.P., Tennen, H., Wagner, J., 2014. Differential item functioning on the Schedule of Racist Events: exploring a modified scale for samples with black and white participants. Ethn Dis 24, 406–412.

Feldt, T., Rantanen, J., Hyvönen, K., Mäkikangas, A., Huhtala, M., Pihlajasaari, P., Kinnunen, U., 2014. The 9-item Bergen Burnout Inventory: factorial validity across organizations and measurements of longitudinal data. Ind Health 52, 102–112. doi:10.2486/indhealth.2013-0059

Feng-Bin, L., Yong-Xing, J., Yu-Hang, W., Zheng-Kun, H., Xin-Lin, C., 2014. Translation and psychometric evaluation of the Chinese version of functional digestive disorders quality of life questionnaire. Dig. Dis. Sci. 59, 390–420. doi:10.1007/s10620-013-2897-1

Fergus, T.A., Valentiner, D.P., Kim, H.-S., McGrath, P.B., 2014. The Social Interaction Anxiety Scale (SIAS) and the Social Phobia Scale (SPS): a comparison of two short-form versions. Psychol Assess 26, 1281–1291. doi:10.1037/a0037313

Ferro, M.A., Boyle, M.H., Scott, J.G., Dingle, K., 2014. The child behavior checklist and youth self-report in adolescents with epilepsy: testing measurement invariance of the attention and thought problems subscales. Epilepsy Behav 31, 34–42. doi:10.1016/j.yebeh.2013.11.009

Finch, W.H., Hernandez Finch, M.E., 2014. Differential item functioning analysis using a multilevel Rasch mixture model: investigating the impact of disability status and receipt of testing accommodations. J Appl Meas 15, 133–151.

Fong, T.C.T., Ho, R.T.H., 2014. Testing gender invariance of the hospital anxiety and depression scale using the classical approach and Bayesian approach. Qual Life Res 23, 1421–1426. doi:10.1007/s11136-013-0594-3

Fonseca-Pedrero, E., Compton, M.T., Tone, E.B., Ortuño-Sierra, J., Paino, M., Fumero, A., Lemos-Giráldez, S., 2014. Cross-cultural invariance of the factor structure of the Schizotypal Personality Questionnaire across Spanish and American college students. Psychiatry Res. 220(3) doi:10.1016/j.psychres.2014.06.050

Ford, D.C., Merrick, M.T., Parks, S.E., Breiding, M.J., Gilbert, L.K., Edwards, V.J., Dhingra, S.S., Barile, J.P., Thompson, W.W., 2014. Examination of the Factorial Structure of Adverse Childhood Experiences and Recommendations for Three Subscale Scores. Psychol Violence 4, 432–444. doi:10.1037/a0037723

Forero, C.G., Adroher, N.D., Stewart-Brown, S., Castellví, P., Codony, M., Vilagut, G., Mompart, A., Tresseres, R., Colom, J., Castro, J.I., Alonso, J., 2014. Differential item and test functioning methodology indicated that item response bias was not a substantial cause of country differences in mental well-being. J Clin Epidemiol 67, 1364–1374. doi:10.1016/j.jclinepi.2014.06.017

Forget, N.J., Jerosch-Herold, C., Shepstone, L., Higgins, J., 2014. Psychometric evaluation of the Disabilities of the Arm, Shoulder and Hand (DASH) with Dupuytren’s contracture: validity evidence using Rasch modeling. BMC Musculoskelet Disord 15, 361. doi:10.1186/1471-2474-15-361

Forrest, C.B., Bevans, K.B., Pratiwadi, R., Moon, J., Teneralli, R.E., Minton, J.M., Tucker, C.A., 2014. Development of the PROMIS ® pediatric global health (PGH-7) measure. Qual Life Res 23, 1221–1231. doi:10.1007/s11136-013-0581-8

Fournet, N., Roulin, J.-L., Monnier, C., Atzeni, T., Cosnefroy, O., Le Gall, D., Roy, A., 2015. Multigroup confirmatory factor analysis and structural invariance with age of the Behavior Rating Inventory of Executive Function (BRIEF)--French version. Child Neuropsychol 21, 379–398. doi:10.1080/09297049.2014.906569

Frazier, T.W., Ratliff, K.R., Gruber, C., Zhang, Y., Law, P.A., Constantino, J.N., 2014. Confirmatory factor analytic structure and measurement invariance of quantitative autistic traits measured by the social responsiveness scale-2. Autism 18, 31–44. doi:10.1177/1362361313500382

Gabrielli, J., Jackson, Y., Brown, S., 2015. Measurement of Behavioral and Emotional Outcomes of Youth in Foster Care: Investigation of the Roles of Age and Placement Type. J Psychopathol Behav Assess 37, 422–431. doi:10.1007/s10862-014-9464-8

Ghorbani, N., Watson, P.J., Farhadi, M., Chen, Z., 2014. A multi-process model of self-regulation: influences of mindfulness, integrative self-knowledge and self-control in Iran. Int J Psychol 49, 115–122. doi:10.1002/ijop.12033

Gibbons, C.J., Kenning, C., Coventry, P.A., Bee, P., Bundy, C., Fisher, L., Bower, P., 2013a. Development of a multimorbidity illness perceptions scale (MULTIPleS). PLoS ONE 8, e81852. doi:10.1371/journal.pone.0081852

Gibbons, C.J., Thornton, E.W., Ealing, J., Shaw, P.J., Talbot, K., Tennant, A., Young, C.A., UKMND-QoL Group, 2013b. Assessing social isolation in motor neurone disease: a Rasch analysis of the MND Social Withdrawal Scale. J. Neurol. Sci. 334, 112–118. doi:10.1016/j.jns.2013.08.002

Gilder, D.A., Gizer, I.R., Lau, P., Ehlers, C.L., 2014. Item response theory analyses of DSM-IV and DSM-5 stimulant use disorder criteria in an American Indian community sample. Drug Alcohol Depend 135, 29–36. doi:10.1016/j.drugalcdep.2013.10.010

Gomes, R.R. de F.M., Batista, J.R., Ceccato, M. das G.B., Kerr, L.R.F.S., Guimarães, M.D.C., 2014. HIV/AIDS knowledge among men who have sex with men: applying the item response theory. Rev Saude Publica 48, 206–215. doi: 10.1590/S0034-8910.2014048004911

Gomez, R., 2014a. Teacher ratings of ODD symptoms: measurement equivalence across Malaysian Malay, Chinese and Indian children. Asian J Psychiatr 8, 52–55. doi:10.1016/j.ajp.2013.10.017

Gomez, R., 2014b. Malaysian parent and teacher ratings of the oppositional defiant disorder symptoms: measurement invariance and parent-teacher agreement. Asian J Psychiatr 11, 35–38. doi:10.1016/j.ajp.2014.05.002

Gomez, R., McLaren, S., Sharp, M., Smith, C., Hearn, K., Turner, L., 2015. Evaluation of the bifactor structure of the dispositional hope scale. J Pers Assess 97, 191–199. doi:10.1080/00223891.2014.938158

Gomez, R., Summers, M., Summers, A., Wolf, A., Summers, J., 2014. Depression Anxiety Stress Scales-21: measurement and structural invariance across ratings of men and women. Assessment 21, 418–426. doi:10.1177/1073191113514106

Gómez-Simón, I., Penelo, E., de la Osa, N., 2014. Factor structure and measurement invariance of the Difficulties Emotion Regulation Scale (DERS) in Spanish adolescents. Psicothema 26, 401–408. doi:10.7334/psicothema2013.324

González-Díez, Z., Orue Sola, I., Calvete Zumalde, E., Riskind, J.H., 2014. Psychometric properties of the Looming Maladaptive Style Questionnaire (LMSQ-R) in young Spanish adults. Psicothema 26, 260–266. doi:10.7334/psicothema2013.206

González-Pérez, M., Susi, R., Antona, B., Barrio, A., González, E., 2014. The Computer-Vision Symptom Scale (CVSS17): development and initial validation. Invest. Ophthalmol. Vis. Sci. 55, 4504–4511. doi:10.1167/iovs.13-13818

Gothwal, V.K., Bagga, D.K., Sumalini, R., 2014. Rasch validation of the PHQ-9 in people with visual impairment in South India. J Affect Disord 167, 171–177. doi:10.1016/j.jad.2014.06.019

Granero, R., Penelo, E., Stinchfield, R., Fernández-Aranda, F., Aymamí, N., Gómez-Peña, M., Fagundo, A.B., Sauchelli, S., Islam, M.A., Menchón, J.M., Jiménez-Murcia, S., 2014. Contribution of illegal acts to pathological gambling diagnosis: DSM-5 implications. J Addict Dis 33, 41–52. doi:10.1080/10550887.2014.882730

Grassi-Oliveira, R., Cogo-Moreira, H., Salum, G.A., Brietzke, E., Viola, T.W., Manfro, G.G., Kristensen, C.H., Arteche, A.X., 2014. Childhood Trauma Questionnaire (CTQ) in Brazilian samples of different age groups: findings from confirmatory factor analysis. PLoS ONE 9, e87118. doi:10.1371/journal.pone.0087118

Guertler, D., Broda, A., Bischof, A., Kastirke, N., Meerkerk, G.-J., John, U., Meyer, C., Rumpf, H.-J., 2014. Factor structure of the compulsive internet use scale. Cyberpsychol Behav Soc Netw 17, 46–51. doi:10.1089/cyber.2013.0076

Guttersrud, O., Dalane, J.Ø., Pettersen, S., 2014. Improving measurement in nutrition literacy research using Rasch modelling: examining construct validity of stage-specific “critical nutrition literacy” scales. Public Health Nutr 17, 877–883. doi:10.1017/S1368980013000530

Hahn, E.A., DeWalt, D.A., Bode, R.K., Garcia, S.F., DeVellis, R.F., Correia, H., Cella, D., PROMIS Cooperative Group, 2014a. New English and Spanish social health measures will facilitate evaluating health determinants. Health Psychol 33, 490–499. doi:10.1037/hea0000055

Hahn, E.A., Kallen, M.A., Jacobs, E.A., Ganschow, P.S., Garcia, S.F., Burns, J.L., 2014b. English-Spanish equivalence of the Health Literacy Assessment Using Talking Touchscreen Technology (Health LiTT). J Health Commun 19 Suppl 2, 285–301. doi:10.1080/10810730.2014.936567

Halberstadt, A.G., Dunsmore, J.C., Bryant, A., Parker, A.E., Beale, K.S., Thompson, J.A., 2013. Development and validation of the Parents’ Beliefs About Children’s Emotions Questionnaire. Psychol Assess 25, 1195–1210. doi:10.1037/a0033695

Haltigan, J.D., Leerkes, E.M., Wong, M.S., Fortuna, K., Roisman, G.I., Supple, A.J., O’Brien, M., Calkins, S.D., Plamondon, A., 2014. Adult attachment states of mind: measurement invariance across ethnicity and associations with maternal sensitivity. Child Dev 85, 1019–1035.

doi: 10.1111/cdev.12180

Haroz, E.E., Ybarra, M.L., Eaton, W.W., 2014. Psychometric evaluation of a self-report scale to measure adolescent depression: the CESDR-10 in two national adolescent samples in the United States. J Affect Disord 158, 154–160. doi:10.1016/j.jad.2014.02.009

Harris-Haywood, S., Goode, T., Gao, Y., Smith, K., Bronheim, S., Flocke, S.A., Zyzanski, S., 2014. Psychometric evaluation of a cultural competency assessment instrument for health professionals. Med Care 52, e7–e15. doi:10.1097/MLR.0b013e31824df149

Hartley, C.M., Barroso, N., Rey, Y., Pettit, J.W., Bagner, D.M., 2014. Factor structure and psychometric properties of english and spanish versions of the edinburgh postnatal depression scale among Hispanic women in a primary care setting. J Clin Psychol 70, 1240–1250. doi:10.1002/jclp.22101

Hauck-Filho, N., Teixeira, M.A.P., 2014. Revisiting the psychometric properties of the Levenson self-report psychopathy scale. J Pers Assess 96, 459–464. doi:10.1080/00223891.2013.865196

He, Q., Glas, C.A.W., Veldkamp, B.P., 2014. Assessing impact of differential symptom functioning on post-traumatic stress disorder (PTSD) diagnosis. Int J Methods Psychiatr Res 23, 131–141. doi:10.1002/mpr.1417

Hecimovich, M.D., Styles, I., Volet, S.E., 2014. Development and psychometric evaluation of scales to measure professional confidence in manual medicine: a Rasch measurement approach. BMC Res Notes 7, 338. doi:10.1186/1756-0500-7-338

Hill, B., Kahn, M., Pallant, J., Williams, G., 2014. Assessment of the internal construct validity of the revised High-Level Mobility Assessment Tool for traumatic orthopaedic injuries. Clin Rehabil 28, 491–498. doi:10.1177/0269215513502798

Hobbs, M.J., Anderson, T.M., Slade, T., Andrews, G., 2014. Relationship between measurement invariance and age-related differences in the prevalence of generalized anxiety disorder. J Affect Disord 152–154, 306–312. doi:10.1016/j.jad.2013.09.030

Hoertel, N., Peyre, H., Wall, M.M., Limosin, F., Blanco, C., 2014. Examining sex differences in DSM-IV borderline personality disorder symptom expression using Item Response Theory (IRT). J Psychiatr Res 59, 213–219. doi:10.1016/j.jpsychires.2014.08.019

Horan, J.M., Brown, J.L., Jones, S.M., Aber, J.L., 2015. Assessing invariance across sex and race/ethnicity in measures of youth psychopathic characteristics. Psychol Assess 27, 657–668. doi:10.1037/pas0000043

Hsu, K., Iwamoto, D.K., 2014. Testing for Measurement Invariance in the Conformity to Masculine Norms-46 Across White and Asian American College Men: Development and Validity of the CMNI-29. Psychol Men Masc 15, 397–406. doi:10.1037/a0034548

Huang, F.L., Cornell, D.G., Konold, T.R., 2015. Aggressive Attitudes in Middle Schools: A Factor Structure and Criterion-Related Validity Study. Assessment 22, 497–512. doi:10.1177/1073191114551016

Hung, M., Hon, S.D., Franklin, J.D., Kendall, R.W., Lawrence, B.D., Neese, A., Cheng, C., Brodke, D.S., 2014. Psychometric properties of the PROMIS physical function item bank in patients with spinal disorders. Spine 39, 158–163. doi:10.1097/BRS.0000000000000097

Iliceto, P., Fino, E., Sabatello, U., Candilera, G., 2014. Personality and suicidal ideation in the elderly: factorial invariance and latent means structures across age. Aging Ment Health 18, 792–800. doi:10.1080/13607863.2014.880404

Inchausti, F., Prieto, G., Delgado, A.R., 2014. Rasch analysis of the Spanish version of the Mindful Attention Awareness Scale (MAAS) in a clinical sample. Rev Psiquiatr Salud Ment 7, 32–41. doi:10.1016/j.rpsm.2013.07.003

Ismail, S.Y., Timmerman, L., Timman, R., Luchtenburg, A.E., Smak Gregoor, P.J.H., Nette, R.W., van den Dorpel, R.M.A., Zuidema, W.C., Weimar, W., Massey, E.K., Busschbach, J.J.V., 2013. A psychometric analysis of the Rotterdam Renal Replacement Knowledge-Test (R3K-T) using item response theory. Transpl. Int. 26, 1164–1172. doi:10.1111/tri.12188

Janssens, A., Goossens, L., Van Den Noortgate, W., Colpin, H., Verschueren, K., Van Leeuwen, K., 2015. Parents’ and Adolescents’ Perspectives on Parenting: Evaluating Conceptual Structure, Measurement Invariance, and Criterion Validity. Assessment 22, 473–489. doi:10.1177/1073191114550477

Janulis, P., 2014. Improving measurement of injection drug risk behavior using item response theory. Am J Drug Alcohol Abuse 40, 143–150. doi:10.3109/00952990.2013.848212

Kalibatseva, Z., Leong, F.T.L., Ham, E.H., 2014. A symptom profile of depression among Asian Americans: is there evidence for differential item functioning of depressive symptoms? Psychol Med 44, 2567–2578. doi:10.1017/S0033291714000130

Kamody, R.C., Berlin, K.S., Hains, A.A., Kichler, J.C., Davies, W.H., Diaz-Thomas, A.M., Ferry, R.J., 2014. Assessing measurement invariance of the diabetes stress questionnaire in youth with type 1 diabetes. J Pediatr Psychol 39, 1138–1148. doi:10.1093/jpepsy/jsu076

Kim, G., Sellbom, M., Ford, K.-L., 2014. Race/ethnicity and measurement equivalence of the Everyday Discrimination Scale. Psychol Assess 26, 892–900. doi:10.1037/a0036431

Kim, S.Y., Wang, Y., Weaver, S.R., Shen, Y., Wu-Seibold, N., Liu, C.H., 2014. Measurement equivalence of the language-brokering scale for Chinese American adolescents and their parents. J Fam Psychol 28, 180–192. doi:10.1037/a0036030

King-Kallimanis, B.L., Kenny, R.A., Savva, G.M., 2014. Factor structure for the frailty syndrome was consistent across Europe. J Clin Epidemiol 67, 1008–1015. doi:10.1016/j.jclinepi.2014.05.002

Klages, U., Erbe, C., Sandru, S.D., Brüllman, D., Wehrbein, H., 2015. Psychosocial impact of dental aesthetics in adolescence: validity and reliability of a questionnaire across age-groups. Qual Life Res 24, 379–390. doi:10.1007/s11136-014-0767-8

Kool, M.B., van de Schoot, R., López-Chicheri García, I., Mewes, R., Da Silva, J.A.P., Vangronsveld, K., Wismeijer, A.A.J., Lumley, M.A., van Middendorp, H., Bijlsma, J.W.J., Crombez, G., Rief, W., Geenen, R., 2014. Measurement invariance of the Illness Invalidation Inventory (3*I) across language, rheumatic disease and gender. Ann. Rheum. Dis. 73, 551–556. doi:10.1136/annrheumdis-2012-201807

Koopmans, L., Bernaards, C.M., Hildebrandt, V.H., van Buuren, S., van der Beek, A.J., de Vet, H.C.W., 2014. Improving the Individual Work Performance Questionnaire using Rasch analysis. J Appl Meas 15, 160–175. doi: 10.1136/oemed-2013-101717.51

Krahn, G.L., Horner-Johnson, W., Hall, T.A., Roid, G.H., Andresen, E.M., Fujiura, G.T., Nosek, M.A., Cardinal, B.J., Drum, C.E., Suzuki, R., Peterson, J.J., 2014. Development and psychometric assessment of the function-neutral health-related quality of life measure. Am J Phys Med Rehabil 93, 56–74. doi:10.1097/PHM.0b013e3182a517e6

Kreiner, S., Christensen, K.B., 2014. Analyses of model fit and robustness. A new look at the PISA scaling model underlying ranking of countries according to reading literacy. Psychometrika 79, 210–231. doi:10.1007/s11336-013-9347-z

Krieger, T., Zimmermann, J., Huffziger, S., Ubl, B., Diener, C., Kuehner, C., Grosse Holtforth, M., 2014. Measuring depression with a well-being index: further evidence for the validity of the WHO Well-Being Index (WHO-5) as a measure of the severity of depression. J Affect Disord 156, 240–244. doi:10.1016/j.jad.2013.12.015

Küçükdeveci, A.A., Ataman, Ş., Genç, A., Kutlay, Ş., Elhan, A.H., Őztuna, D., Tennant, A., 2015. Adaptation and validation of the Ankylosing Spondylitis Work Instability Scale (AS-WIS) for use in Turkey. Rheumatol. Int. 35, 125–131. doi:10.1007/s00296-014-3050-y

Kwakkenbos, L., Willems, L.M., Baron, M., Hudson, M., Cella, D., van den Ende, C.H.M., Thombs, B.D., Canadian Scleroderma Research Group, 2014. The comparability of English, French and Dutch scores on the Functional Assessment of Chronic Illness Therapy-Fatigue (FACIT-F): an assessment of differential item functioning in patients with systemic sclerosis. PLoS ONE 9, e91979. doi:10.1371/journal.pone.0091979

La Greca, A.M., Ingles, C.J., Lai, B.S., Marzo, J.C., 2015. Social Anxiety Scale for Adolescents: factorial invariance across gender and age in Hispanic American adolescents. Assessment 22, 224–232. doi:10.1177/1073191114540749

Lai, J.-S., Zelko, F., Krull, K.R., Cella, D., Nowinski, C., Manley, P.E., Goldman, S., 2014. Parent-reported cognition of children with cancer and its potential clinical usefulness. Qual Life Res 23, 1049–1058. doi:10.1007/s11136-013-0548-9

Lampard, A.M., Nishi, A., Baskin, M.L., Carson, T.L., Davison, K.K., 2014. The Activity Support Scale for Multiple Groups (ACTS-MG): Child-reported Physical Activity Parenting in African American and Non-Hispanic White Families. Behav Med 1–8, 112-119 doi:10.1080/08964289.2014.979757

Las Hayas, C., Calvete, E., Gómez del Barrio, A., Beato, L., Muñoz, P., Padierna, J.Á., 2014. Resilience Scale-25 Spanish version: validation and assessment in eating disorders. Eat Behav 15, 460–463. doi:10.1016/j.eatbeh.2014.06.010

Latimer, S., Meade, T., Tennant, A., 2014. Development of item bank to measure deliberate self-harm behaviours: facilitating tailored scales and computer adaptive testing for specific research and clinical purposes. Psychiatry Res 217, 240–247. doi:10.1016/j.psychres.2014.03.015

Lee, J., Huang, Y., Robertson, M.M., Murphy, L.A., Garabet, A., Chang, W.-R., 2014. External validity of a generic safety climate scale for lone workers across different industries and companies. Accid Anal Prev 63, 138–145. doi:10.1016/j.aap.2013.10.013

Lin, X., Li, M., Wang, M., Zuo, Y., Zhu, S., Zheng, Y., Lin, X., Yu, M., Lamoureux, E.L., 2014. Validation of Catquest-9SF questionnaire in a Chinese cataract population. PLoS ONE 9, e103860. doi:10.1371/journal.pone.0103860

Liu, J.D., Chung, P.K., Chen, W.P., 2014. Constraints of recreational sport participation: measurement invariance and latent mean differences across sex and physical activity status. Percept Mot Skills 119, 363–376. doi:10.2466/06.03.PMS.119c24z0

Lucas-Molina, B., Williamson, A.A., Pulido, R., Calderón, S., 2014. Adaptation of the Participant Role Scale (PRS) in a Spanish youth sample: measurement invariance across gender and relationship with sociometric status. J Interpers Violence 29, 2904–2930. doi:10.1177/0886260514527822

Lundgren-Nilsson, A., Dencker, A., Jakobsson, S., Taft, C., Tennant, A., 2014. Construct validity of the Swedish version of the revised piper fatigue scale in an oncology sample--a Rasch analysis. Value Health 17, 360–363. doi:10.1016/j.jval.2014.02.010

Makransky, G., Bilenberg, N., 2014. Psychometric properties of the parent and teacher ADHD Rating Scale (ADHD-RS): measurement invariance across gender, age, and informant. Assessment 21, 694–705. doi:10.1177/1073191114535242

Marc, L.G., Henderson, W.R., Desrosiers, A., Testa, M.A., Jean, S.E., Akom, E.E., 2014. Reliability and validity of the Haitian Creole PHQ-9. J Gen Intern Med 29, 1679–1686. doi:10.1007/s11606-014-2951-5

Marcus, D.K., Zeigler-Hill, V., Mercer, S.H., Norris, A.L., 2014. The psychology of spite and the measurement of spitefulness. Psychol Assess 26, 563–574. doi:10.1037/a0036039

Marra, L.B., Field, C.A., Caetano, R., von Sternberg, K., 2014. Construct validity of the short inventory of problems among Spanish speaking Hispanics. Addict Behav 39, 205–210. doi:10.1016/j.addbeh.2013.09.023

Marsden, J., Eastwood, B., Ali, R., Burkinshaw, P., Chohan, G., Copello, A., Burn, D., Kelleher, M., Mitcheson, L., Taylor, S., Wilson, N., Whiteley, C., Day, E., 2014. Development of the Addiction Dimensions for Assessment and Personalised Treatment (ADAPT). Drug Alcohol Depend 139, 121–131. doi:10.1016/j.drugalcdep.2014.03.018

Matsuda, P.N., Taylor, C.S., Shumway-Cook, A., 2014. Evidence for the validity of the modified dynamic gait index across diagnostic groups. Phys Ther 94, 996–1004. doi:10.2522/ptj.20130294

McGrane, J.A., Butow, P.N., Sze, M., Eisenbruch, M., Goldstein, D., King, M.T., 2014. Assessing the invariance of a culturally competent multi-lingual unmet needs survey for immigrant and Australian-born cancer patients: a Rasch analysis. Qual Life Res 23, 2819–2830. doi:10.1007/s11136-014-0717-5

McManus, I.C., Chis, L., Fox, R., Waller, D., Tang, P., 2014. Implementing statistical equating for MRCP(UK) Parts 1 and 2. BMC Med Educ 14, 204. doi:10.1186/1472-6920-14-204

Memetovic, J., Ratner, P.A., Richardson, C.G., 2014. Gender-based measurement invariance of the substance use risk profile scale. Addict Behav 39, 690–694. doi:10.1016/j.addbeh.2013.10.016

Mercer, S.H., McIntosh, K., Strickland-Cohen, M.K., Horner, R.H., 2014. Measurement invariance of an instrument assessing sustainability of school-based universal behavior practices. Sch Psychol Q 29, 125–137. doi:10.1037/spq0000054

Merz, E.L., Malcarne, V.L., Roesch, S.C., Ko, C.M., Emerson, M., Roma, V.G., Sadler, G.R., 2013. Psychometric properties of Positive and Negative Affect Schedule (PANAS) original and short forms in an African American community sample. J Affect Disord 151, 942–949. doi:10.1016/j.jad.2013.08.011

Miche, M., Elsässer, V.C., Schilling, O.K., Wahl, H.-W., 2014. Attitude toward own aging in midlife and early old age over a 12-year period: examination of measurement equivalence and developmental trajectories. Psychol Aging 29, 588–600. doi:10.1037/a0037259

Mills, S.D., Malcarne, V.L., Fox, R.S., Sadler, G.R., 2014. Psychometric Evaluation of the Brief Acculturation Scale for Hispanics. Hisp J Behav Sci 36, 164–174. doi:10.1177/0739986314526697

Missinne, S., Vandeviver, C., Van de Velde, S., Bracke, P., 2014. Measurement equivalence of the CES-D 8 depression-scale among the ageing population in eleven European countries. Soc Sci Res 46, 38–47. doi:10.1016/j.ssresearch.2014.02.006

Mordeno, I.G., Nalipay, M.J.N., Untalan, J.H.C., Decatoria, J.B., 2014. Examining posttraumatic stress disorder’s latent structure between treatment-seeking and non-treatment-seeking Filipinos. Asian J Psychiatr 11, 28–34. doi:10.1016/j.ajp.2014.05.003

Morean, M.E., DeMartini, K.S., Leeman, R.F., Pearlson, G.D., Anticevic, A., Krishnan-Sarin, S., Krystal, J.H., O’Malley, S.S., 2014. Psychometrically improved, abbreviated versions of three classic measures of impulsivity and self-control. Psychol Assess 26, 1003–1020. doi:10.1037/pas0000003

Morizot, J., 2014. Construct validity of adolescents’ self-reported big five personality traits: importance of conceptual breadth and initial validation of a short measure. Assessment 21, 580–606. doi:10.1177/1073191114524015

Morris, S.B., Huang, J., Zhao, L., Sergent, J.D., Neuhengen, J., 2014. Measurement equivalence of the Empowerment Scale for White and Black persons with severe mental illness. Psychiatr Rehabil J 37, 277–283. doi:10.1037/prj0000069

Morsunbul, U., Crocetti, E., Cok, F., Meeus, W., 2014. Brief report: the Utrecht-Management of Identity Commitments Scale (U-MICS): gender and age measurement invariance and convergent validity of the Turkish version. J Adolesc 37, 799–805. doi:10.1016/j.adolescence.2014.05.008

Mueller, A.E., Segal, D.L., Gavett, B., Marty, M.A., Yochim, B., June, A., Coolidge, F.L., 2015. Geriatric Anxiety Scale: item response theory analysis, differential item functioning, and creation of a ten-item short form (GAS-10). Int Psychogeriatr 27, 1099–1111. doi:10.1017/S1041610214000210

Mulder, H., Hoofs, H., Verhagen, J., van der Veen, I., Leseman, P.P.M., 2014. Psychometric properties and convergent and predictive validity of an executive function test battery for two-year-olds. Front Psychol 5, 733. doi:10.3389/fpsyg.2014.00733

Munyombwe, T., Höfer, S., Fitzsimons, D., Thompson, D.R., Lane, D., Smith, K., Astin, F., 2014. An evaluation of the Minnesota Living with Heart Failure Questionnaire using Rasch analysis. Qual Life Res 23, 1753–1765. doi:10.1007/s11136-013-0617-0

Murphy, C.C., McQueen, A., Bartholomew, L.K., Del Junco, D.J., Coan, S.P., Vernon, S.W., 2013. Factorial validity and invariance of four psychosocial constructs of colorectal cancer screening: does screening experience matter? Cancer Epidemiol. Biomarkers Prev. 22, 2295–2302. doi:10.1158/1055-9965.EPI-13-0565

Ortuño-Sierra, J., Badoud, D., Knecht, F., Paino, M., Eliez, S., Fonseca-Pedrero, E., Debbané, M., 2013. Testing measurement invariance of the schizotypal personality questionnaire-brief scores across Spanish and Swiss adolescents. PLoS ONE 8, e82041. doi:10.1371/journal.pone.0082041

Osman, A., Lamis, D.A., Freedenthal, S., Gutierrez, P.M., McNaughton-Cassill, M., 2014. The multidimensional scale of perceived social support: analyses of internal reliability, measurement invariance, and correlates across gender. J Pers Assess 96, 103–112. doi:10.1080/00223891.2013.838170

Oude Voshaar, M.A.H., ten Klooster, P.M., Glas, C.A.W., Vonkeman, H.E., Taal, E., Krishnan, E., Moens, H.J.B., Boers, M., Terwee, C.B., van Riel, P.L.C.M., van de Laar, M.A.F.J., 2014. Calibration of the PROMIS physical function item bank in Dutch patients with rheumatoid arthritis. PLoS ONE 9, e92367. doi:10.1371/journal.pone.0092367

Ownby, R.L., Acevedo, A., Waldrop-Valverde, D., Jacobs, R.J., 2014. Is the cloze procedure appropriate to evaluate health literacy in older individuals? Age effects in the test of functional health literacy in adults. J Aging Res 2014, 194635. doi:10.1155/2014/194635

Pape, T.L.-B., Mallinson, T., Guernon, A., 2014. Psychometric properties of the disorders of consciousness scale. Arch Phys Med Rehabil 95, 1672–1684. doi:10.1016/j.apmr.2014.04.015

Parker, G., Fletcher, K., McCraw, S., Synnott, H., Friend, P., Mitchell, P.B., Hadzi-Pavlovic, D., 2014. Screening for bipolar disorder: does gender distort scores and case-finding estimates? J Affect Disord 162, 55–60. doi:10.1016/j.jad.2014.03.032

Patterson Silver Wolf Adelv Unegv Waya, D.A., Dulmus, C.N., Maguin, E., Fava, N., 2014. Refining the Evidence-Based Practice Attitude Scale: An Alternative Confirmatory Factor Analysis. Soc Work Res 38, 47–58. doi:10.1093/swr/svu006

Paudel, P., Khadka, J., Burnett, A., Hani, Y., Naduvilath, T., Fricke, T.R., 2015. Papua New Guinea vision-specific quality of life questionnaire: a new patient-reported outcome instrument to assess the impact of impaired vision. Clin. Experiment. Ophthalmol. 43, 202–213. doi:10.1111/ceo.12413

Pauwels, E., Claes, L., Dierckx, E., Debast, I., Van Alphen, S.P.J.B., Rossi, G., Schotte, C., Santens, E., Peuskens, H., 2014. Age neutrality of the young schema questionnaire in patients with a substance use disorder. Int Psychogeriatr 26, 1317–1326. doi:10.1017/S1041610214000519

Pendergast, L.L., Scharf, R.J., Rasmussen, Z.A., Seidman, J.C., Schaefer, B.A., Svensen, E., Tofail, F., Koshy, B., Kosek, M., Rasheed, M.A., Roshan, R., Maphula, A., Shrestha, R., Murray-Kolb, L.E., MAL-ED Network Investigators, 2014. Postpartum depressive symptoms across time and place: structural invariance of the Self-Reporting Questionnaire among women from the international, multi-site MAL-ED study. J Affect Disord 167, 178–186. doi:10.1016/j.jad.2014.05.039

Penelo, E., Negrete, A., Portell, M., Raich, R.M., 2013. Psychometric properties of the Eating Disorder Examination Questionnaire (EDE-Q) and norms for rural and urban adolescent males and females in Mexico. PLoS ONE 8, e83245. doi:10.1371/journal.pone.0083245

Peter, C., Cieza, A., Geyh, S., 2014. Rasch analysis of the General Self-Efficacy Scale in spinal cord injury. J Health Psychol 19, 544–555. doi:10.1177/1359105313475897

Petersen, J.J., Paulitsch, M.A., Hartig, J., Mergenthal, K., Gerlach, F.M., Gensichen, J., 2015. Factor structure and measurement invariance of the Patient Health Questionnaire-9 for female and male primary care patients with major depression in Germany. J Affect Disord 170, 138–142. doi:10.1016/j.jad.2014.08.053

Peyre, H., Hoertel, N., Cortese, S., Acquaviva, E., De Maricourt, P., Limosin, F., Delorme, R., 2014. Attention-deficit/hyperactivity disorder symptom expression: a comparison of individual age at onset using item response theory. J Clin Psychiatry 75, 386–392. doi:10.4088/JCP.13m08638

Pilatti, A., Read, J.P., Vera, B. del V., Caneto, F., Garimaldi, J.A., Kahler, C.W., 2014. The Spanish version of the Brief Young Adult Alcohol Consequences Questionnaire (B-YAACQ): a Rasch model analysis. Addict Behav 39, 842–847. doi:10.1016/j.addbeh.2014.01.026

Pollard, B., Johnston, M., Dixon, D., 2013. Exploring differential item functioning in the SF-36 by demographic, clinical, psychological and social factors in an osteoarthritis population. BMC Musculoskelet Disord 14, 346. doi:10.1186/1471-2474-14-346

Preti, A., Vellante, M., Gabbrielli, M., Lai, V., Muratore, T., Pintus, E., Pintus, M., Sanna, S., Scanu, R., Tronci, D., Corrias, I., Petretto, D.R., Carta, M.G., 2013. Confirmatory factor analysis and measurement invariance by gender, age and levels of psychological distress of the short TEMPS-A. J Affect Disord 151, 995–1002. doi:10.1016/j.jad.2013.08.025

Prieto, G., Nieto, E., 2014. Influence of DIF on differences in performance of Italian and Asian individuals on a reading comprehension test of Spanish as a foreign language (negative emotionality) in Hong Kong. J Appl Meas 15, 176–188.

Puig, A., Yoon, E., Callueng, C., An, S., Lee, S.M., 2014. Burnout syndrome in psychotherapists: a comparative analysis of five nations. Psychol Serv 11, 87–96. doi:10.1037/a0035285

Pula, K., Parks, C.D., Ross, C.F., 2014. Regulatory focus and food choice motives. Prevention orientation associated with mood, convenience, and familiarity. Appetite 78, 15–22. doi:10.1016/j.appet.2014.02.015

Purc-Stephenson, R.J., 2014. The Posttraumatic Growth Inventory: factor structure and invariance among persons with chronic diseases. Rehabil Psychol 59, 10–18. doi:10.1037/a0035353

Randall, M., Imms, C., Carey, L.M., Pallant, J.F., 2014. Rasch analysis of The Melbourne Assessment of Unilateral Upper Limb Function. Dev Med Child Neurol 56, 665–672. doi:10.1111/dmcn.12391

Reddy, L.A., Fabiano, G., Dudek, C.M., Hsu, L., 2013. Development and construct validity of the Classroom Strategies Scale-Observer Form. Sch Psychol Q 28, 317–341. doi:10.1037/spq0000043

Reilly, E.E., Anderson, L.M., Schaumberg, K., Anderson, D.A., 2014. Gender-based differential item functioning in common measures of body dissatisfaction. Body Image 11, 206–209. doi:10.1016/j.bodyim.2014.02.001

Rendas-Baum, R., Yang, M., Varon, S.F., Bloudek, L.M., DeGryse, R.E., Kosinski, M., 2014. Validation of the Headache Impact Test (HIT-6) in patients with chronic migraine. Health Qual Life Outcomes 12, 117. doi:10.1186/s12955-014-0117-0

Riazi, A., Aspden, T., Jones, F., 2014. Stroke Self-efficacy Questionnaire: a Rasch-refined measure of confidence post stroke. J Rehabil Med 46, 406–412. doi:10.2340/16501977-1789

Ribeiro, J.D., Witte, T.K., Van Orden, K.A., Selby, E.A., Gordon, K.H., Bender, T.W., Joiner, T.E., 2014. Fearlessness about death: the psychometric properties and construct validity of the revision to the acquired capability for suicide scale. Psychol Assess 26, 115–126. doi:10.1037/a0034858

Rice, K.G., Richardson, C.M.E., Tueller, S., 2014. The short form of the revised almost perfect scale. J Pers Assess 96, 368–379. doi:10.1080/00223891.2013.838172

Richards, E.A., McDonough, M.H., Edwards, N.E., Lyle, R.M., Troped, P.J., 2013. Development and psychometric testing of the Dogs and WalkinG Survey (DAWGS). Res Q Exerc Sport 84, 492–502. doi:10.1080/02701367.2013.839935

Rodríguez-Carvajal, R., de Rivas, S., Herrero, M., Moreno-Jiménez, B., van Dierendonck, D., 2014. Leading people positively: cross-cultural validation of the Servant Leadership Survey (SLS). Span J Psychol 17, E63. doi:10.1017/sjp.2014.73

Rutherford, C., Nixon, J., Brown, J.M., Lamping, D.L., Cano, S.J., 2014. Using mixed methods to select optimal mode of administration for a patient-reported outcome instrument for people with pressure ulcers. BMC Med Res Methodol 14, 22. doi:10.1186/1471-2288-14-22

Sakaluk, J.K., Todd, L.M., Milhausen, R., Lachowsky, N.J., Undergraduate Research Group In Sexuality URGiS, 2014. Dominant heterosexual sexual scripts in emerging adulthood: conceptualization and measurement. J Sex Res 51, 516–531. doi:10.1080/00224499.2012.745473

Samuel, D.B., South, S.C., Griffin, S.A., 2015. Factorial invariance of the Five-Factor Model Rating Form across gender. Assessment 22, 65–75. doi:10.1177/1073191114536772

Sargent-Cox, K.A., Rippon, M., Burns, R.A., 2014. Measuring anxiety about aging across the adult lifespan. Int Psychogeriatr 26, 135–145. doi:10.1017/S1041610213001798

Sayegh, P., Knight, B.G., 2014. Functional assessment and neuropsychiatric inventory questionnaires: measurement invariance across hispanics and non-Hispanic whites. Gerontologist 54, 375–386. doi:10.1093/geront/gnt026

Schwartz, S.J., Benet-Martínez, V., Knight, G.P., Unger, J.B., Zamboanga, B.L., Des Rosiers, S.E., Stephens, D.P., Huang, S., Szapocznik, J., 2014. Effects of language of assessment on the measurement of acculturation: measurement equivalence and cultural frame switching. Psychol Assess 26, 100–114. doi:10.1037/a0034717

Seabra, A.C., Malina, R.M., Parker, M., Seabra, A., Brustad, R., Maia, J.A., Fonseca, A.M., 2014. Validation and factorial invariance of children’s attraction to physical activity (CAPA) scale in Portugal. Eur J Sport Sci 14, 384–391. doi:10.1080/17461391.2013.828777

Shadel, W.G., Edelen, M.O., Tucker, J.S., Stucky, B.D., Hansen, M., Cai, L., 2014a. Development of the PROMIS coping expectancies of smoking item banks. Nicotine Tob. Res. 16 Suppl 3, S202-211. doi:10.1093/ntr/ntu040

Shadel, W.G., Edelen, M.O., Tucker, J.S., Stucky, B.D., Hansen, M., Cai, L., 2014b. Development of the PROMIS nicotine dependence item banks. Nicotine Tob. Res. 16 Suppl 3, S190-201. doi:10.1093/ntr/ntu032

Sharp, C., Michonski, J., Steinberg, L., Fowler, J.C., Frueh, B.C., Oldham, J.M., 2014. An investigation of differential item functioning across gender of BPD criteria. J Abnorm Psychol 123, 231–236. doi:10.1037/a0035637

Sheu, H.-B., Chong, S.S., Chen, H.-F., Lin, W.-C., 2014. Well-being of Taiwanese and Singaporean college students: cross-cultural validity of a modified social cognitive model. J Couns Psychol 61, 447–460. doi:10.1037/cou0000018

Shono, Y., Grenard, J.L., Ames, S.L., Stacy, A.W., 2014. Application of item response theory to tests of substance-related associative memory. Psychol Addict Behav 28, 852–862. doi:10.1037/a0035877

Sierakowska, M., Sierakowski, S., Sierakowska, J., Horton, M., Ndosi, M., 2015. Developing the Polish Educational Needs Assessment Tool (Pol-ENAT) in rheumatoid arthritis and systemic sclerosis: a cross-cultural validation study using Rasch analysis. Qual Life Res 24, 721–733. doi:10.1007/s11136-014-0805-6

Skriner, L.C., Chu, B.C., 2014. Cross-ethnic measurement invariance of the SCARED and CES-D in a youth sample. Psychol Assess 26, 332–337. doi:10.1037/a0035092

Skule, C., Ulleberg, P., Dallavara Lending, H., Berge, T., Egeland, J., Brennen, T., Landrø, N.I., 2014. Depressive symptoms in people with and without alcohol abuse: factor structure and measurement invariance of the Beck Depression Inventory (BDI-II) across groups. PLoS ONE 9, e88321. doi:10.1371/journal.pone.0088321

Smith, E.A., Miller, J.A., Newsome, V., Sofolahan, Y.A., Airhihenbuwa, C.O., 2014. Measuring HIV/AIDS-Related Stigma Across South Africa: A Versatile and Multidimensional Scale. Health Educ Behav 41, 387–391. doi:10.1177/1090198113515245

Spaapen, D.L., Waters, F., Brummer, L., Stopa, L., Bucks, R.S., 2014. The emotion regulation questionnaire: validation of the ERQ-9 in two community samples. Psychol Assess 26, 46–54. doi:10.1037/a0034474

Spangenberg, L., Zenger, M., Rief, W., Brähler, E., Glaesmer, H., 2014. Assessing modern health worries: dimensionality and factorial invariance across age and sex of the Modern Health Worries Scale in a general population sample. J Health Psychol 19, 1302–1308. doi:10.1177/1359105313488980

Specht, J., Luhmann, M., Geiser, C., 2014. On the consistency of personality types across adulthood: latent profile analyses in two large-scale panel studies. J Pers Soc Psychol 107, 540–556. doi:10.1037/a0036863

Spinhoven, P., Penninx, B.W., Hickendorff, M., van Hemert, A.M., Bernstein, D.P., Elzinga, B.M., 2014. Childhood Trauma Questionnaire: factor structure, measurement invariance, and validity across emotional disorders. Psychol Assess 26, 717–729. doi:10.1037/pas0000002

Starosta, A.J., Berghoff, C.R., Earleywine, M., 2015. Factor structure and gender stability in the multidimensional condom attitudes scale. Assessment 22, 374–384. doi:10.1177/1073191114547887

Steca, P., Monzani, D., Greco, A., Chiesi, F., Primi, C., 2015. Item response theory analysis of the life orientation test-revised: age and gender differential item functioning analyses. Assessment 22, 341–350. doi:10.1177/1073191114544471

Sterling, K.L., Ford, K.H., Park, H., McAlister, A.L., 2014. Scales of smoking-related self-efficacy, beliefs, and intention: assessing measurement invariance among intermittent and daily high school smokers. Am J Health Promot 28, 310–315. doi:10.4278/ajhp.121009-QUAN-490

Stevanovic, D., Jafari, P., 2015. A cross-cultural study to assess measurement invariance of the KIDSCREEN-27 questionnaire across Serbian and Iranian children and adolescents. Qual Life Res 24, 223–230. doi:10.1007/s11136-014-0754-0

Stevanovic, D., Urbán, R., Atilola, O., Vostanis, P., Singh Balhara, Y.P., Avicenna, M., Kandemir, H., Knez, R., Franic, T., Petrov, P., 2015. Does the Strengths and Difficulties Questionnaire - self report yield invariant measurements across different nations? Data from the International Child Mental Health Study Group. Epidemiol Psychiatr Sci 24, 323–334. doi:10.1017/S2045796014000201

Stucky, B.D., Edelen, M.O., Sherbourne, C.D., Eberhart, N.K., Lara, M., 2014a. Developing an item bank and short forms that assess the impact of asthma on quality of life. Respir Med 108, 252–263. doi:10.1016/j.rmed.2013.12.008

Stucky, B.D., Edelen, M.O., Tucker, J.S., Shadel, W.G., Cerully, J., Kuhfeld, M., Hansen, M., Cai, L., 2014b. Development of the PROMIS negative psychosocial expectancies of smoking item banks. Nicotine Tob. Res. 16 Suppl 3, S232-240. doi:10.1093/ntr/ntt282

Suárez-Álvarez, J., Pedrosa, I., García-Cueto, E., Muñiz, J., 2014. Screening enterprising personality in youth: an empirical model. Span J Psychol 17, E60. doi:10.1017/sjp.2014.61

Sumner, J.A., Pietrzak, R.H., Danielson, C.K., Adams, Z.W., Ruggiero, K.J., 2014. Elucidating dimensions of posttraumatic stress symptoms and their functional correlates in disaster-exposed adolescents. J Psychiatr Res 59, 85–92. doi:10.1016/j.jpsychires.2014.09.003

Tadić, V., Cooper, A., Cumberland, P., Lewando-Hundt, G., Rahi, J.S., Vision-related Quality of Life Group, 2013. Development of the functional vision questionnaire for children and young people with visual impairment: the FVQ_CYP. Ophthalmology 120, 2725–2732. doi:10.1016/j.ophtha.2013.07.055

Taylor, J.M., 2015. Psychometric analysis of the Ten-Item Perceived Stress Scale. Psychol Assess 27, 90–101. doi:10.1037/a0038100

Teale, E.A., Munyombwe, T.M., Young, J.B., 2013. Scaling properties of the subjective index of physical and social outcome after stroke in a study population unselected by age. Arch Phys Med Rehabil 94, 2448–2455. doi:10.1016/j.apmr.2013.08.243

Teresi, J.A., Ocepek-Welikson, K., Ramirez, M., Kleinman, M., Ornstein, K., Siu, A., 2015. Evaluation of measurement equivalence of the Family Satisfaction with the End-of-Life Care in an ethnically diverse cohort: tests of differential item functioning. Palliat Med 29, 83–96. doi:10.1177/0269216314545802

Terluin, B., Smits, N., Miedema, B., 2014. The English version of the four-dimensional symptom questionnaire (4DSQ) measures the same as the original Dutch questionnaire: a validation study. Eur J Gen Pract 20, 320–326. doi:10.3109/13814788.2014.905826

Thewes, B., Zachariae, R., Christensen, S., Nielsen, T., Butow, P., 2015. The Concerns About Recurrence Questionnaire: validation of a brief measure of fear of cancer recurrence amongst Danish and Australian breast cancer survivors. J Cancer Surviv 9, 68–79. doi:10.1007/s11764-014-0383-1

Thomas, S., Kersten, P., Thomas, P.W., 2015. The Multiple Sclerosis-Fatigue Self- Efficacy (MS-FSE) scale: initial validation. Clin Rehabil 29, 376–387. doi:10.1177/0269215514543702

Tian, F., Ni, P., Mulcahey, M.J., Hambleton, R.K., Tulsky, D., Haley, S.M., Jette, A.M., 2014. Tracking functional status across the spinal cord injury lifespan: linking pediatric and adult patient-reported outcome scores. Arch Phys Med Rehabil 95, 2078–2085.e15. doi:10.1016/j.apmr.2014.05.023

Tian, L., Han, M., Huebner, E.S., 2014. Preliminary development of the adolescent students’ basic psychological needs at school scale. J Adolesc 37, 257–267. doi:10.1016/j.adolescence.2014.01.005

Tomás, I., Marsh, H.W., González-Romá, V., Valls, V., Nagengast, B., 2014. Testing measurement invariance across Spanish and English versions of the physical self-description questionnaire: an application of exploratory structural equation modeling. J Sport Exerc Psychol 36, 179–188. doi:10.1123/jsep.2013-0070

Treloar, H.R., Martens, M.P., McCarthy, D.M., 2014. Testing measurement invariance of the protective behavioral strategies scale in college men and women. Psychol Assess 26, 307–313. doi:10.1037/a0034471

Tsai, J., Elhai, J.D., Pietrzak, R.H., Hoff, R.A., Harpaz-Rotem, I., 2014. Comparing four competing models of depressive symptomatology: a confirmatory factor analytic study of 986,647 U.S. veterans. J Affect Disord 165, 166–169. doi:10.1016/j.jad.2014.04.075

Tsang, S., Piquero, A.R., Cauffman, E., 2014. An examination of the Psychopathy Checklist: Youth Version (PCL:YV) among male adolescent offenders: an item response theory analysis. Psychol Assess 26, 1333–1346. doi:10.1037/a0037500

Tucker, J.S., Shadel, W.G., Edelen, M.O., Stucky, B.D., Kuhfeld, M., Hansen, M., Cai, L., 2014a. Development of the PROMIS Social Motivations for Smoking item banks. Nicotine Tob. Res. 16 Suppl 3, S241-249. doi:10.1093/ntr/ntt283

Tucker, J.S., Shadel, W.G., Edelen, M.O., Stucky, B.D., Li, Z., Hansen, M., Cai, L., 2014b. Development of the PROMIS positive emotional and sensory expectancies of smoking item banks. Nicotine Tob. Res. 16 Suppl 3, S212-222. doi:10.1093/ntr/ntt281

Twiss, J., McKenna, S.P., 2015. Comparing the impact of psoriasis and atopic dermatitis on quality of life: co-calibration of the PSORIQoL and QoLIAD. Qual Life Res 24, 105–113. doi:10.1007/s11136-014-0630-y

Tylka, T.L., Wood-Barcalow, N.L., 2015. The Body Appreciation Scale-2: item refinement and psychometric evaluation. Body Image 12, 53–67. doi:10.1016/j.bodyim.2014.09.006

Tyser, A.R., Beckmann, J., Franklin, J.D., Cheng, C., Hon, S.D., Wang, A., Hung, M., 2014. Evaluation of the PROMIS physical function computer adaptive test in the upper extremity. J Hand Surg Am 39, 2047–2051.e4. doi:10.1016/j.jhsa.2014.06.130

Van den Broeck, W., Hofmans, J., Cooremans, S., Staels, E., 2014. Factorial validity and measurement invariance across intelligence levels and gender of the overexcitabilities questionnaire-II (OEQ-II). Psychol Assess 26, 55–68. doi:10.1037/a0034475

van der Wal, M.B.A., Tuinebreijer, W.E., Lundgren-Nilsson, Å., Middelkoop, E., van Zuijlen, P.P.M., 2014. Differential item functioning in the Observer Scale of the POSAS for different scar types. Qual Life Res 23, 2037–2045. doi:10.1007/s11136-014-0637-4

van der Zwaard, B.C., Terwee, C.B., Roddy, E., Terluin, B., van der Horst, H.E., Elders, P.J.M., 2014. Evaluation of the measurement properties of the Manchester foot pain and disability index. BMC Musculoskelet Disord 15, 276. doi:10.1186/1471-2474-15-276

Varni, J.W., Thissen, D., Stucky, B.D., Liu, Y., Magnus, B., Quinn, H., Irwin, D.E., DeWitt, E.M., Lai, J.-S., Amtmann, D., Gross, H.E., DeWalt, D.A., 2014. PROMIS® Parent Proxy Report Scales for children ages 5-7 years: an item response theory analysis of differential item functioning across age groups. Qual Life Res 23, 349–361. doi:10.1007/s11136-013-0439-0

Veale, J.F., 2014. Edinburgh Handedness Inventory - Short Form: a revised version based on confirmatory factor analysis. Laterality 19, 164–177. doi:10.1080/1357650X.2013.783045

Vinding, G.R., Christensen, K.B., Esmann, S., Olesen, A.B., Jemec, G.B.E., 2013. Quality of life in non-melanoma skin cancer--the skin cancer quality of life (SCQoL) questionnaire. Dermatol Surg 39, 1784–1793. doi:10.1111/dsu.12353

Vleeschouwer, M., Schubart, C.D., Henquet, C., Myin-Germeys, I., van Gastel, W.A., Hillegers, M.H.J., van Os, J.J., Boks, M.P.M., Derks, E.M., 2014. Does assessment type matter? A measurement invariance analysis of online and paper and pencil assessment of the Community Assessment of Psychic Experiences (CAPE). PLoS ONE 9, e84011. doi:10.1371/journal.pone.0084011

Wanders, R.B.K., Wardenaar, K.J., Kessler, R.C., Penninx, B.W.J.H., Meijer, R.R., de Jonge, P., 2015. Differential reporting of depressive symptoms across distinct clinical subpopulations: what DIFference does it make? J Psychosom Res 78, 130–136. doi:10.1016/j.jpsychores.2014.08.014

Wang, K.T., Wei, M., Zhao, R., Chuang, C.-C., Li, F., 2015. The Cross-Cultural Loss Scale: development and psychometric evaluation. Psychol Assess 27, 42–53. doi:10.1037/pas0000027

Wang, Y.-C., Deutscher, D., Yen, S.-C., Werneke, M.W., Mioduski, J.E., 2014. The self-report fecal incontinence and constipation questionnaire in patients with pelvic-floor dysfunction seeking outpatient rehabilitation. Phys Ther 94, 273–288. doi:10.2522/ptj.20130062

Watt, T., Barbesino, G., Bjorner, J.B., Bonnema, S.J., Bukvic, B., Drummond, R., Groenvold, M., Hegedüs, L., Kantzer, V., Lasch, K.E., Marcocci, C., Mishra, A., Netea-Maier, R., Ekker, M., Paunovic, I., Quinn, T.J., Rasmussen, Å.K., Russell, A., Sabaretnam, M., Smit, J., Törring, O., Zivaljevic, V., Feldt-Rasmussen, U., 2015. Cross-cultural validity of the thyroid-specific quality-of-life patient-reported outcome measure, ThyPRO. Qual Life Res 24, 769–780. doi:10.1007/s11136-014-0798-1

Watt, T., Groenvold, M., Hegedüs, L., Bonnema, S.J., Rasmussen, Å.K., Feldt-Rasmussen, U., Bjorner, J.B., 2014. Few items in the thyroid-related quality of life instrument ThyPRO exhibited differential item functioning. Qual Life Res 23, 327–338. doi:10.1007/s11136-013-0462-1

Weller, K.E., Greene, G.W., Redding, C.A., Paiva, A.L., Lofgren, I., Nash, J.T., Kobayashi, H., 2014. Development and validation of green eating behaviors, stage of change, decisional balance, and self-efficacy scales in college students. J Nutr Educ Behav 46, 324–333. doi:10.1016/j.jneb.2014.01.002

White, S.W., Lerner, M.D., McLeod, B.D., Wood, J.J., Ginsburg, G.S., Kerns, C., Ollendick, T., Kendall, P.C., Piacentini, J., Walkup, J., Compton, S., 2015. Anxiety in youth with and without autism spectrum disorder: examination of factorial equivalence. Behav Ther 46, 40–53. doi:10.1016/j.beth.2014.05.005

Wolf, T., Zimprich, D., 2015. Differences in the use of autobiographical memory across the adult lifespan. Memory 23, 1238–1254. doi:10.1080/09658211.2014.971815

Wong, Q.J.J., Moulds, M.L., 2014. An examination of the measurement equivalence of the Brief Fear of Negative Evaluation scale across individuals who identify with an asian ethnicity and individuals who identify with a European ethnicity. Assessment 21, 713–722. doi:10.1177/1073191114528570

Woo, S.E., Chernyshenko, O.S., Longley, A., Zhang, Z.-X., Chiu, C.-Y., Stark, S.E., 2014. Openness to Experience: its lower level structure, measurement, and cross-cultural equivalence. J Pers Assess 96, 29–45. doi:10.1080/00223891.2013.806328

Wu, P.-C., Huang, T.-W., 2014. Gender-related invariance of the Beck Depression Inventory II for Taiwanese adolescent samples. Assessment 21, 218–226. doi:10.1177/1073191112441243

Yan, Y., Wu, W., Strunk, R.C., Garbutt, J., 2014. Use of factor analysis models to evaluate measurement invariance property of the Asthma Control Questionnaire (ACQ). Qual Life Res 23, 509–513. doi:10.1007/s11136-013-0474-x

Yap, S.C.Y., Donnellan, M.B., Schwartz, S.J., Kim, S.Y., Castillo, L.G., Zamboanga, B.L., Weisskirch, R.S., Lee, R.M., Park, I.J.K., Whitbourne, S.K., Vazsonyi, A.T., 2014. Investigating the structure and measurement invariance of the Multigroup Ethnic Identity Measure in a multiethnic sample of college students. J Couns Psychol 61, 437–446. doi:10.1037/a0036253

Yekaninejad, M.S., Pakpour, A.H., Tadakamadla, J., Kumar, S., Mosavi, S.H., Fridlund, B., Bottomley, A., Aaronson, N.K., 2015. Oral-health-related quality of life in patients with cancer: cultural adaptation and the psychometric testing of the Persian version of EORTC QLQ-OH17. Support Care Cancer 23, 1215–1224. doi:10.1007/s00520-014-2468-9

Yount, K.M., VanderEnde, K., Zureick-Brown, S., Anh, H.T., Schuler, S.R., Minh, T.H., 2014a. Measuring attitudes about intimate partner violence against women: the ATT-IPV scale. Demography 51, 1551–1572. doi:10.1007/s13524-014-0297-6

Yount, K.M., VanderEnde, K., Zureick-Brown, S., Minh, T.H., Schuler, S.R., Anh, H.T., 2014b. Measuring attitudes about women’s recourse after exposure to intimate partner violence: the ATT-RECOURSE scale. J Interpers Violence 29, 1579–1605. doi:10.1177/0886260513511536

Yuan, C., Wei, C., Wang, J., Qian, H., Lev, E.L., Yuan, A., Li, H., Hinds, P.S., 2014. Testing measurement invariance of the Chinese version of the Strategies Used by Patients to Promote Health among patients with cancer. J Nurs Meas 22, 184–200. doi : /10.1891/1061-3749.22.2.184

Zhang, C.-Q., Si, G., Chung, P.-K., Du, M., Terry, P.C., 2014. Psychometric properties of the Brunel Mood Scale in Chinese adolescents and adults. J Sports Sci 32, 1465–1476. doi:10.1080/02640414.2014.898184

Zhong, Q., Gelaye, B., Fann, J.R., Sanchez, S.E., Williams, M.A., 2014. Cross-cultural validity of the Spanish version of PHQ-9 among pregnant Peruvian women: a Rasch item response theory analysis. J Affect Disord 158, 148–153. doi:10.1016/j.jad.2014.02.012

Zucoloto, M.L., Maroco, J., Campos, J. a. D.B., 2014. Psychometric Properties of the Oral Health Impact Profile and New Methodological Approach. J. Dent. Res. 93, 645–650. doi:10.1177/0022034514533798
