# Supplementary material for: Differential Item Functioning (DIF) in composite health measurement scale: Recommendations for characterizing DIF with meaningful consequences within the Rasch model framework
Source: PLoS One. 2019 Apr 9;14(4):e0215073. doi: 10.1371/journal.pone.0215073 (PMC6456214; doi:10.1371/journal.pone.0215073)
Supplement: S1 Box — (DOCX) [file pone.0215073.s004.docx]

**S1 - Box**: Recommendations for characterizing relevant differential item functioning in composite measurement scales used in health research studies within the Rasch model framework.

**Recommendation 1**

*Distinguish between uniform, balanced non-uniform and unbalanced non-uniform DIF.*

**Recommendation 2**

*When statistically significant DIF is found, its effect-size should be estimated as the group difference in the item location parameter(s).*

**Recommendation 3**

*Due to the presence of statistically significant DIF:*

*- If the scale has been modified, provide information on how it has been modified*

*- If the scale has not been modified, provide recommendations on how to take the presence of DIF with meaningful consequences into account when using the scale in practice, i.e. when:*

🢝 *Statistically significant uniform DIF is found in more than 25% of the items within a dimension*

*🢝 Statistically significant uniform DIF is found with an effect-size higher than 0.25 logit*

🢝 *Statistically significant unbalanced non-uniform DIF is found*

**Recommendation 4**

*If* *several items exhibit statistically significant DIF within the dimension but in opposite direction, assess its effects at the dimension level empirically.*
